# Cellular Adaptation to Mechanical Stress Emerges via Cell Shrinkage Triggered by Nonlinear Calcium Elevation

**DOI:** 10.1002/advs.202503659

**Published:** 2025-07-18

**Authors:** Zhengyan Wang, Zihan Li, Lei Wang, Haitao Zhang, Xiaoshan Yang, Lili Bao, Geng Dou, Lili Ren, Yajing Fu, Lan Li, Shengkai Gong, Yang Zhou, Feng Ding, Lu Yu, Haotian Luo, Yao Liu, Fuyang Zhang, Hui Yu, Siying Liu, Xueming Liu, Fulan Wei, Shiyu Liu

**Affiliations:** ^1^ Department of Orthodontics School and Hospital of Stomatology Cheeloo College of Medicine Shandong University & Shandong Key Laboratory of Oral Tissue Regeneration & Shandong Engineering Research Center of Dental Materials and Oral Tissue Regeneration & Shandong Provincial Clinical Research Center for Oral Diseases Jinan 250012 China; ^2^ State Key Laboratory of Oral & Maxillofacial Reconstruction and Regeneration National Clinical Research Center for Oral Diseases Shaanxi Key Laboratory of Stomatology Department of Oral Biology School of Stomatology The Fourth Military Medical University Xi'an 710032 China; ^3^ State Key Laboratory of Oral & Maxillofacial Reconstruction and Regeneration National Clinical Research Center for Oral Diseases Shaanxi International Joint Research Center for Oral Diseases Center for Tissue Engineering School of Stomatology The Fourth Military Medical University Xi'an 710032 China; ^4^ School of Artificial Intelligence and Automation The MOE Engineering Research Center of Autonomous Intelligent Unmanned Systems the Key Laboratory of Image Processing Huazhong University of Science and Technology Wuhan 430074 China; ^5^ Department of Stomatology Shandong Provincial Hospital Affiliated to Shandong First Medical University Jinan 250021 China; ^6^ Department of Paediatric Dentistry School of Stomatology China Medical University Shenyang 110002 China; ^7^ Department of Cardiology Xijing Hospital The Fourth Military Medical University Xi'an 710032 China; ^8^ Wuhan Children's Hospital: Wuhan Women and Children Medical Care Center Tongji Medical College Huazhong University of Science and Technology Wuhan 430010 China; ^9^ State Key Laboratory of Military Stomatology and National Clinical Research Center for Oral Diseases and Shaanxi Clinical Research Center for Oral Diseases Department of Orthodontics School of Stomatology The Fourth Military Medical University Xi'an 710032 China

**Keywords:** adaptation, cholesterol, extracellular vesicles, mechanical force, positive feedback

## Abstract

Organisms constantly encounter unpredictable environmental perturbations, necessitating adaptation to maintain homeostasis. However, the fundamental principles by which organisms identify specific cues and transition to an adaptive state remain unclear. Here, using a mouse mechanical ventilation model and a cell stretch model, it is found that the cellular adaptation to mechanical stress can be induced by applying low amplitude stretches to cells, and demonstrate that the adaptation emerges once a defined stretch threshold is reached. This adaptive state is marked by transient cell shrinkage and reduced membrane tension. Mechanistically, guided by a mathematical model of intracellular Ca^2+^ dynamics, it is found that when stretch reaches a critical amplitude, it induces Ca^2+^‐dependent positive feedback, leading to nonlinear Ca^2+^ elevation. This activates scramblase Anoctamin‐6, promoting extracellular vesicle‐mediated membrane cholesterol efflux. The reduction in membrane cholesterol subsequently activates volume‐regulated anion channels, leading to cell shrinkage and the establishment of mechanical adaptation. These findings reveal a threshold‐dependent mechanism for mechanical adaptation emergence, and propose a promising strategy to develop targeted interventions in mechanical stress‐related disorders.

## Graphical abstract

Here, a decision‐making mechanism underlying mechanical adaptation is demonstrated. This study demonstrates that cellular adaptation to mechanical stress can be induced by applying low‐amplitude stretch to cells. When the stretch reaches a critical amplitude threshold, it triggers Ca^2^⁺‐dependent positive feedback, which induces cholesterol efflux and activation of volume‐regulated anion channels, leading to cell shrinkage and the emergence of mechanical adaptation.

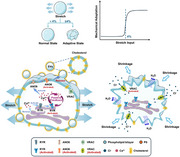

## 1 Introduction

Organisms are continually exposed to unpredictable environmental perturbations, requiring mechanisms of adaptation to maintain homeostasis. Adaptation is defined as the ability to withstand perturbations and establish a new functionally steady state that confers enhanced tolerance to subsequent more severe challenges.^[^ ^1^ ^]^ Accumulating evidence suggests that biological systems develop adaptive responses to stress through low‐intensity preconditioning such as mild cold exposure^[^ ^2^ ^]^ or brief ischemia,^[^ ^3^, ^4^, ^5^ ^]^ thereby enhancing tolerance to subsequent severe stress. While mechanical forces represent ubiquitous physiological stimuli, current researches focus primarily on acute adaptive mechanisms for immediate damage mitigation,^[^ ^6^, ^7^ ^]^ whether sublethal mechanical preconditioning induces adaptive states remains unknown.

Despite the recognized importance of adaptation, the fundamental principles by which organisms identify specific environmental cues and transition into adaptive states remain poorly understood. In many biological systems, state transitions are threshold‐dependent, maintaining stability until perturbations exceed critical levels.^[^ ^8^, ^9^ ^]^ This is illustrated by the action potential, generated when a stimulus changes the membrane potential to a threshold,^[^ ^10^ ^]^ and spore germination, initiated when the inner membrane reaches a defined electrochemical potential.^[^ ^11^ ^]^ Additionally, the occurrences of the first vertebrate heartbeat,^[^ ^12^ ^]^ collective organs regeneration,^[^ ^13^ ^]^ and self‐antigens induced autoimmune responses^[^ ^14^ ^]^ are also governed by specific thresholds and follow all‐or‐none responses. These examples show the nonlinearity in biological responses to perturbations. Compared with linearity, nonlinearity refers to the fact that the output of a system is not proportional to the input, that is, a small change in the input can lead to a large change in the output.^[^ ^15^ ^]^ The nonlinearity characterizes the basic design principle and complex features of biological systems. The understanding of nonlinear biological processes and identification of critical thresholds at which states switch is crucial for the development of precise intervention strategies. However, the role of nonlinearity in adaptation emergence remains to be explored.

Mechanical forces profoundly impact physiological and pathological processes,^[^ ^16^, ^17^ ^]^ including development, regeneration,^[^ ^18^ ^]^ wound healing, fibrosis^[^ ^19^, ^20^ ^]^ and weightlessness‐induced bone loss.^[^ ^21^ ^]^ Emerging evidence suggests that cells adapt to mechanical stress through processes such as nuclear softening^[^ ^6^ ^]^ or caveolae flattening.^[^ ^7^ ^]^ However, it remains unclear whether these adaptive behaviors can evolve into stable states of cellular adaptation that protect against subsequent mechanical challenges. Here, using a mouse mechanical ventilation model and an in vitro cell stretch model, we find the cellular adaptation to mechanical stress can be induced by applying low amplitude stretch to cells, and demonstrate that the adaptation emerges once a defined stretch threshold is reached. This adaptive state is marked by transient cell shrinkage and reduced membrane tension. Guided by a mathematical model of intracellular Ca^2+^ dynamics, we show that when stretch reaches a critical threshold, it induces a positive feedback loop of intracellular Ca^2+^ elevation, triggering volume‐regulated anion channel (VRAC)‐mediated adaptive cell volume reduction and shrinkage.

This study reveals a nonlinear decision‐making mechanism underlying adaptation to mechanical stress, defining the threshold that establishes a sharp decision boundary between the normal state and the adaptive state. Additionally, our findings suggest that the nonlinear emergence of adaptation may be a general feature across various forms of environmental stress, as observed in cellular responses to heat and hypo‐osmotic stress (see Figure S4, Supporting Information). Thus, the understanding of biological nonlinear behavior not only yield insights into the design principles of organisms but also have theoretical implications to biomedical therapy.

## 2 Results

### 2.1 Mild Ventilation that Reaches Critical Threshold Activates Pulmonary Mechanical Adaptation

**Figure 1:**
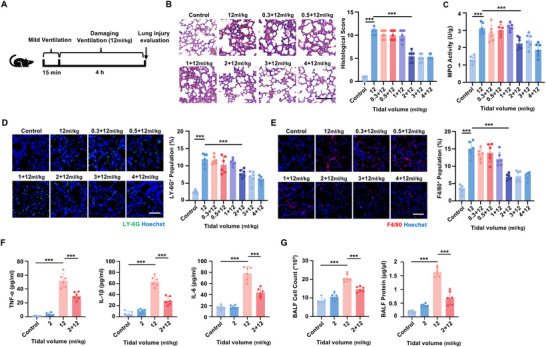
Mild ventilation that reaches critical threshold activates pulmonary mechanical adaptation. A) Schematic diagram of the experimental procedure. To evaluate the effects of mild low tidal volume ventilation on alleviating lung injury caused by damaging high tidal volume ventilation, we conducted varying mild ventilations at or below 4 ml kg^−1^ for 15 min before damaging ventilation at 12 ml kg^−1^ for 4 h. Mice from the untreated control group, the 12 ml kg^−1^ ventilation group, and 12 ml kg^−1^ ventilation with pretreatments with mild ventilation pretreatments at or below 4 ml kg^−1^ were then subjected for further analysis. B) Hematoxylin and eosin (H&E) staining of representative lung section and the associated histological scores assessed by inflammatory infiltration, alveolar wall thickening, microhemorrhages, and alveolar overinflation. Scale bar, 100 µm. n  =  7 mice. C) Myeloperoxidase (MPO) activity of lung tissues. n = 6 mice. D) Representative fluorescence images of the neutrophils marker LY‐6G (green) detected by confocal laser scanning microscope (CLSM) and the percentage of neutrophils infiltration in lung tissues. Scale bar, 100 µm. n  =  6 mice. E) Representative fluorescence images of the macrophage marker F4/80 (red) detected by CLSM and the percentage of macrophages infiltration in lung tissues. Scale bar, 100 µm. n  =  6 mice. F) Concentrations of TNF‐α, IL‐1β, and IL‐6 in bronchoalveolar lavage fluid (BALF) measured by ELISA after ventilation. n = 6 mice. G) Cell counts and protein concentration in BALF from mice following ventilation. n  =  6 mice. Data are presented as mean ± s.d. Statistical significance was assessed by one‐way analysis of variance (ANOVA) with Tukey's post hoc test (C‐G) and Kruskal‐Wallis test (B).

Lung tissues undergo dilation during respiration.^[^ ^22^ ^]^ Under certain pathological conditions, alveolar epithelial cells are exposed to harmful mechanical stress.^[^ ^23^ ^]^ To assess the lung's potential adaptation to mechanical force, we established a mouse model of mechanical ventilation. Mechanical ventilation is a crucial life‐saving intervention for patients with acute respiratory distress syndrome (ARDS). However, it carries a significant risk of causing ventilator‐induced lung injury (VILI) by damaging pulmonary epithelial cells.^[^ ^24^, ^25^, ^26^ ^]^ To investigate whether mild low‐tidal‐volume ventilation could induce mechanical adaptation to VILI induced by damaging high‐tidal‐volume ventilation, we first confirmed that mild ventilation at 0.3‐4 ml kg^−1^ causes no significant injury in lung tissues (Figure S1A,B, Supporting Information). Subsequently, varying mild ventilation was applied respectively before damaging high‐tidal‐volume ventilation at 12 ml kg^−1^ (**Figure** **1**A). Remarkably, the results showed that mild ventilation pretreatment, when reaching a certain critical threshold (2 ml kg^−1^), effectively mitigated lung injury caused by subsequent damaging ventilation at 12 ml kg^−1^. Specifically, as the tidal volume increased, mild ventilation at or above 2 ml kg^−1^ resulted in significantly lower histological injury scores (Figure 1B), as evidenced by preserved alveolar structure, reduced hemorrhage, and diminished alveolar wall thickness. Furthermore, the mild ventilation also lowered myeloperoxidase (MPO) activity, a key indicator of neutrophil activation (Figure 1C). We also observed reductions in neutrophil (Figure 1D) and macrophage infiltration (Figure 1E), along with reduced concentration of inflammatory mediators such as TNF‐α, IL‐1β, and IL‐6 in bronchoalveolar lavage fluid (BALF) (Figure 1F). Notably, pretreatment of ventilation at 2 ml kg^−1^ led to lower cell counts and protein level in BALF, suggesting a restoration of alveolar‐capillary barrier function and cell integrity that was compromised by damaging ventilation at 12 ml kg^−1^ (Figure 1G). These findings demonstrate that mechanical adaptation to damaging ventilation requires mild ventilation to reach critical thresholds.

### 2.2 Mechanical Adaptation to Damaging High‐Amplitude Stretch is Achieved by Cell Shrinkage in a Nonlinear Pattern

**Figure 2:**
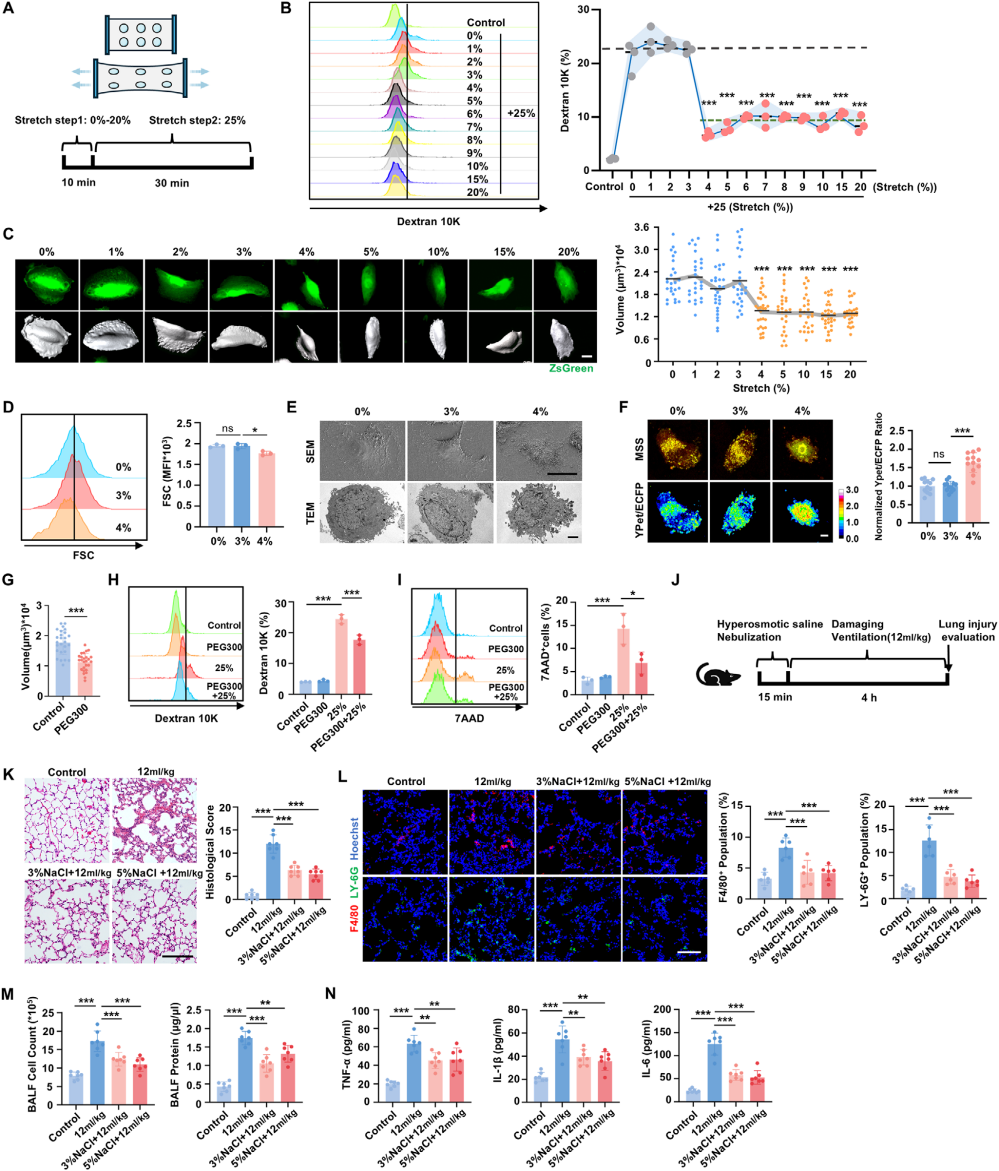
Mechanical adaptation to damaging high‐amplitude stretch is achieved by cell shrinkage in a nonlinear pattern. A) The cell stretch procedure was conducted using a stretch device. Varying mild stretches were conducted respectively prior to the damaging stretch at the amplitude of 25%. B) Representative histograms of Dextran 10K‐FITC penetration in BEAS‐2b cells detected by flow cytometry, with quantitative analysis. n  =  3 biologically independent samples. C) Representative fluorescence images and corresponding 3D images of ZsGreen‐transfected cells captured by CLSM and 3D reconstructed by Imaris software, with quantification of cell volume calculated by Imaris. Scale bar, 10 µm. n > 25 cells per group from three independent experiments. D) Cell volume detected by forward scatter (FSC) on flow cytometry, n  =  3 biologically independent samples. E) Representative transmission electron microscopy (TEM) images and scanning electron microscope (SEM) images of cells. Scale bar for SEM images, 30 µm, for TEM images, 2 µm. n  =  3 biologically independent samples. F) Representative fluorescence images of MSS‐transfected cells and YPet/ECFP emission ratio indicated by the color bar. Scale bar, 10 µm. n  =  12 biologically independent samples. G) Volume of BEAS‐2b cells under hyper‐osmotic compression with 1% PEG300. n > 25 cells per group from three independent experiments. H,I) Representative histograms of Dextran 10K‐FITC penetration (H) and 7AAD staining (I) in BEAS‐2b cells treated with 1% PEG300 prior to 25% stretch and quantitative analysis. n  =  3 biologically independent samples. J) Schematic diagram of the experimental procedure. K–N) Evaluation of the protective effects of hyperosmotic nebulization pretreatment on lung injury induced by damaging ventilation. K) Representative H&E staining and the corresponding histological scores. Scale bar, 100 µm. n  =  7 mice. L) Representative fluorescence images of F4/80^+^ macrophage (red) and LY‐6G^+^ neutrophils (green) in lung tissue detected by CLSM and the quantification of macrophages and neutrophils infiltration. Scale bar, 100 µm. n  =  6 mice. M) Cell counts and protein concentration in BALF. n  =  7 mice. N) TNF‐α, IL‐1β and IL‐6 concentrations in BALF measured by ELISA. n = 7 mice. Data are presented as the mean ± s.d. Statistical significance was assessed by one‐way ANOVA with Tukey's post hoc test (B, D, F, H‐I, K‐N) and Kruskal‐Wallis test (C) and unpaired two‐tailed Student's t‐test (G).

Since mechanical adaptation was observed at the tissue level, we next sought to determine whether similar adaptation exists at the cellular level. A uniaxial cell stretch system was used to apply varying stretch amplitudes (**Figure** **2**A). Bronchial epithelial cells (BEAS‐2b) were exposed to stretches ranging from 1% to 25% for 10 min (Figure S2A, Supporting Information). Dextran 10K‐FITC (MW 10000) was utilized as a penetration marker to assess cell membrane integrity and cell damage. A 25% stretch amplitude resulted in cell damage in 23% of the cells (Figure S2A, Supporting Information) and was selected as the damaging stretch amplitude for subsequent experiments. Mild stretches of varying amplitudes were then applied prior to the 25% damaging stretch. Interestingly, Dextran 10K penetration quantification revealed that the percentage of damaged cells did not gradually decrease as the prior stretch amplitude increased, but instead decreased abruptly in a nonlinear pattern at a critical threshold of 4% (Figure 2B). Beyond this threshold, further increases in prior stretch amplitude did not result in additional reductions in the percentage of damaged cells. Similarly, mild stretch provided a protective effect against damaging stretch in a nonlinear pattern in mouse type II alveolar epithelial cells (Figure S3A, Supporting Information). These findings demonstrate that cells acquire mechanical adaptation in a nonlinear response to mild mechanical stress, mirroring the adaptation observed in the lung tissue in vivo.

Given the observation of mechanical adaptation emergence at both tissue and cellular levels following a nonlinear pattern, we explored whether this might represent a generalized principle whereby cells gain adaptation in response to other forms of stress. To test this hypothesis, we investigated the adaptation of three different cell types when exposed to heat or hypo‐osmotic stress, both of which are common environmental stimuli.^[^ ^27^, ^28^ ^]^ Initially, we assessed the percentage of damaged cells following exposure to graded high‐temperature treatments and selected the temperature at which nearly 50% of the cells were damaged as the damaging heat (Figure S4A,C,E, Supporting Information). Next, we examined whether prior exposure to mild heat stress could induce adaptation to damaging heat stress. Consistent with our earlier findings, mild heat stress reaching a critical temperature threshold significantly reduced cell damage caused by subsequent damaging heat stress in all three cell types (Figure S4B,D,F). Similarly, mild hypo‐osmotic stress, once it reached a critical threshold, attenuated cell damage induced by damaging hypo‐osmotic stress (Figure S4G–L, Supporting Information). These findings suggest that the nonlinear emergence of adaptation may be a generalizable feature across various forms of environmental stress.

Next, we aimed to identify the cellular state that confers mechanical adaptation following stretch. Recognizing that cell volume fluctuates in response to external physical stimuli, such as matrix stiffness^[^ ^29^ ^]^ and compression,^[^ ^30^ ^]^ we measured changes in cell volume and morphology after applying stretches of varying amplitudes. Cell volume was assessed by fluorescently labeling the body, followed by confocal microscopy to visualize the 3D boundaries of the cells. We found that cell volume remained unchanged at stretch amplitudes below 4% but significantly decreased at or above 4% (Figure 2C), consistent with the threshold for mild stretch‐induced mechanical adaptation. Flow cytometry further validated these findings, showing a marked difference in cell volume between 3% and 4% stretch (Figure 2D). In parallel, similar results were also observed in alveolar epithelial cells. Cell volume remained stable during stretches below 3% amplitude but significantly decreased above 3% (Figure S3B,C, Supporting Information), aligning with the mechanical adaptation threshold (Figure S3A, Supporting Information). Additionally, transmission and scanning electron microscopy images revealed that compared with 3% stretch, 4% stretch induced cell shrinkage and the formation of microvilli‐like structures and folds in the plasma membrane (Figure 2E), potentially creating a membrane reservoir to buffer against damage from the high‐amplitude stretch. Moreover, using a membrane stress sensor (MSS),^[^ ^31^, ^32^ ^]^ we detected that 4% stretch resulted in decreased cell membrane tension compared to 3% stretch and untreated control cells, as indicated by a higher YPet/ECFP ratio (Figure 2F). This further confirmed that mild mechanical stretch induces membrane redundancy, enabling cells to withstand damaging mechanical stretch. Furthermore, cells that underwent shrinkage following stretch returned to their original size after 12 h (Figure S5A, Supporting Information), with membrane tension also recovering over this period (Figure S5B, Supporting Information) indicating that the adaptive state is reversible. Consequently, we propose that nonlinear cell shrinkage and reduced membrane tension induced by mild stretch help buffer against damaging stretch, thereby alleviating cell damage.

To further elucidate the role of cell shrinkage in mechanical adaptation, we investigated whether the direct application of hyperosmotic conditions to pre‐compress cell volume and induce shrinkage could generate mechanical adaptation. BEAS‐2b cells or alveolar epithelial cells were pretreated in a hyperosmotic condition containing 1% PEG300 to reduce cell volume before exposure to 25% stretch (Figure 2G). Consistent with our hypothesis, hyperosmotic pretreatment alleviated 25%‐stretch‐induced cell damage, as evidenced by reduced Dextran 10K penetration (Figure 2H; Figure S3D, Supporting Information) and a decreased 7AAD^+^ cell ratio (Figure 2I; Figure S3E, Supporting Information) by flow cytometry. Additionally, a 40 mm sorbitol hyperosmotic condition was applied to reduce cell volume before 25% stretch (Figure S5C, Supporting Information), further confirming that cell shrinkage enhances cellular adaptation to mechanical stress (Figure S5D, Supporting Information).

In vivo, mild ventilation in mice also led to the shrinkage of bronchial epithelial cells (Figure S5E, Supporting Information). Building on this, we investigated the potential protective effects of hyperosmotic nebulization pretreatment against lung injury induced by damaging ventilation (Figure 2J). Nebulization pretreatment with 3% or 5% hyperosmotic saline effectively alleviated subsequent ventilation‐induced tissue damage, as reflected by lower histological scores (Figure 2K) and reduced macrophage and neutrophil infiltration (Figure 2L). Furthermore, mice that received nebulization pretreatment showed a significant reduction in alveolocapillary permeability (Figure 2M) and lower levels of inflammatory mediators such as TNF‐α, IL‐1β, and IL‐6 in BALF (Figure 2N). These results demonstrate that hyperosmotic pretreatment‐induced cell shrinkage mitigates both cellular and tissue damage caused by mechanical stretch.

Collectively, our in vivo and in vitro findings suggest that nonlinear cell shrinkage is crucial for enduring damaging mechanical stretch and represents a key characteristic of the mechanically adaptive state.

### 2.3 Nonlinear Cell Shrinkage is Triggered by an Intracellular Ca^2+^‐Dependent Positive Feedback Loop

**Figure 3:**
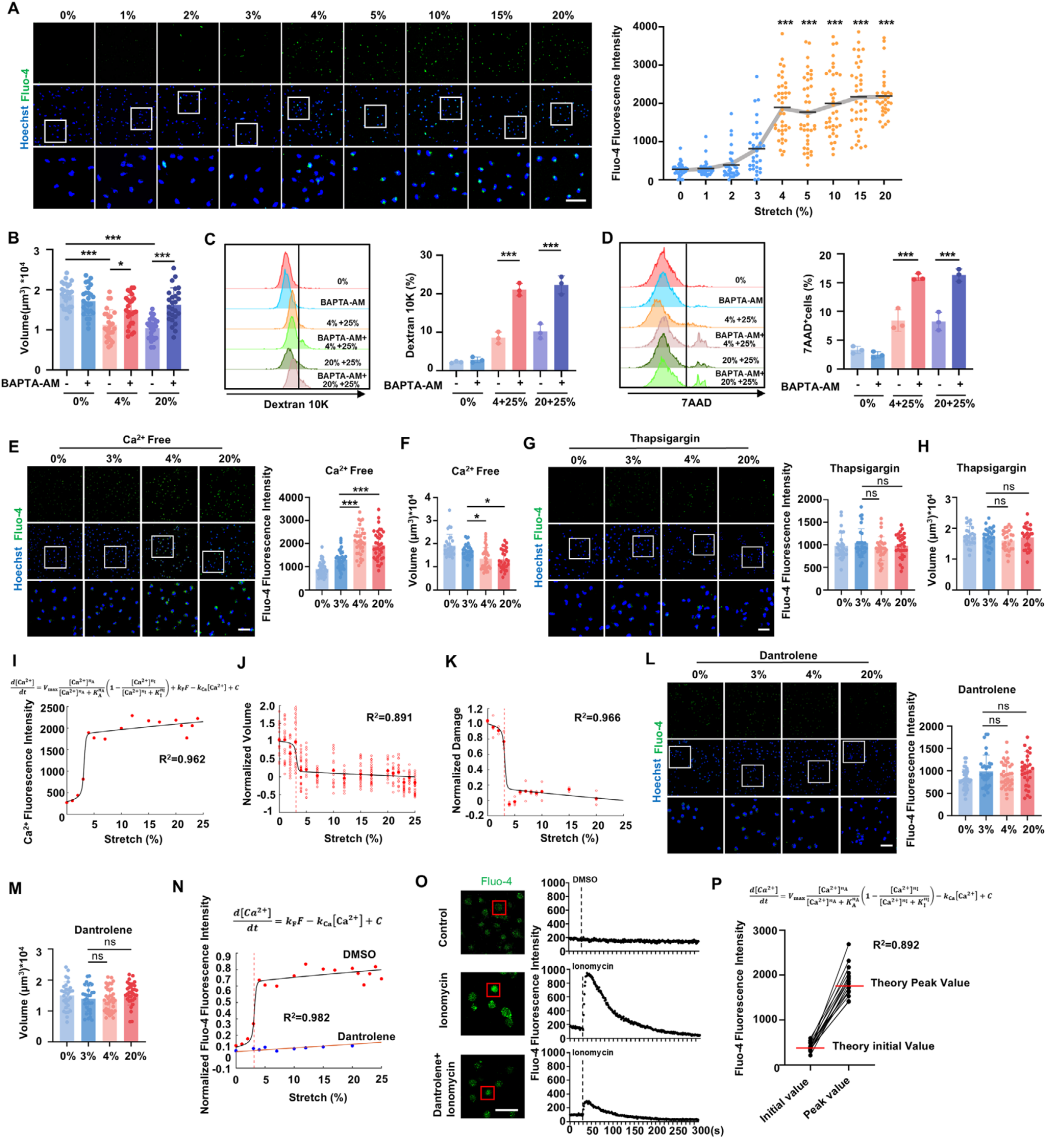
Nonlinear cell shrinkage is triggered by an intracellular Ca^2+^‐dependent positive feedback loop. A) Representative fluorescence images of Ca^2+^ sensor dye Fluo‐4‐AM and quantitative fluorescence intensity in cells detected by CLSM after stretches of varying amplitudes. n > 30 cells per group from three independent experiments. Scale bar, 50 µm. B) The volume of BEAS‐2b cells pretreated with Ca^2+^ chelator BAPTA‐AM prior to 4% or 20% stretch, analyzed by Imaris. n > 25 cells per group from three independent experiments. C,D) Representative histograms of Dextran 10K‐FITC penetration (C) and 7AAD staining (D) in BEAS‐2b cells pretreated with BAPTA‐AM following the process of 4%+25% or 20%+25% stretch, with quantitative analysis. n  =  3 biologically independent samples. E) Representative fluorescence images of Fluo‐4 and quantitative fluorescence intensity of cells stretched in Ca^2+^ free medium. n > 30 cells per group from three independent experiments. Scale bar, 50 µm. F) The volume of BEAS‐2b cells stretched in Ca^2+^ free medium. n > 25 cells per group from three independent experiments. G) Representative fluorescence images of Fluo‐4 and quantitative fluorescence intensity of stretched cells pretreated with Thapsigargin to empty ER Ca^2+^ stores. n > 30 cells per group from three independent experiments. Scale bar, 50 µm. H) The volume of stretched cells pretreated with Thapsigargin. n > 25 cells per group from three independent experiments. I) A mathematical model of varying stretch amplitude‐Fluo‐4 fluorescence intensity fits the experimental data. J,K) Fit of normalized experimental data of stretch amplitude‐cell volume (J) and stretch amplitude‐damaged cell percentage (K) to the normalized stretch amplitude‐Fluo‐4 fluorescence intensity model. L) Representative fluorescence images of Fluo‐4 and quantitative fluorescence intensity of stretched cells pretreated with RYR inhibitor Dantrolene. n > 30 cells per group from three independent experiments. Scale bar, 50 µm. M) The volume of stretched BEAS‐2b cells pretreated with Dantrolene. Scale bar, 50 µm. n > 30 cells per group from three independent experiments. N) Fit of normalized stretch amplitude‐Fluo‐4 fluorescence intensity to the normalized model without Hill's equation part, under Dantrolene pretreatment conditions. O) Real‐time Ca^2+^ dynamics in cells treated with ionomycin with or without Dantrolene pretreatment. Scale bar, 50 µm. P) The fit of initial and peak Fluo‐4 fluorescence intensity to the stretch amplitude‐Ca^2+^ model without the “force‐induced Ca^2+^ elevation” part, under ionomycin treatment. Data are presented as the mean ± s.d. Statistical significance was assessed by one‐way ANOVA with Tukey's post hoc test (B‐E, H, M) and Kruskal‐Wallis test (A, F, G, L).

We further investigated the fundamental mechanisms triggering nonlinear cellular shrinkage in response to mechanical stretch. Intracellular Ca^2+^ elevation, a significant response to mechanical stretch,^[^ ^6^, ^33^ ^]^ prompted us to examine the role of Ca^2+^ signaling in cell volume reduction. Fluorescence imaging and quantification analysis revealed that as the stretch amplitude increased, intracellular Ca^2+^ levels rose nonlinearly once a threshold amplitude of 4% was reached (**Figure** **3**A), reflecting the pattern observed in stretch‐induced cell volume changes. The consistency between volumetric responses and the Ca^2+^ response under stretch was further corroborated in alveolar epithelial cells (Figure S6A, Supporting Information). Moreover, chelating intracellular calcium with BAPTA‐AM inhibited the stretch‐induced cell volume reduction at both 4% and 20% stretch in BEAS‐2B cells (Figure 3B) and at 3% stretch in alveolar epithelial cells (Figure S6B, Supporting Information), and concurrently impaired mechanical adaptation, this was evidenced by increased cell damage under a 25% stretch shown by Dextran 10K penetration (Figure 3C; Figure S6C, Supporting Information) and 7AAD^+^ cell ratio (Figure 3D; Figure S6D, Supporting Information). These findings confirm that both cell shrinkage and the development of mechanical adaptation are Ca^2+^‐dependent processes. We then sought to identify the source of increased intracellular Ca^2+^. Removal of Ca^2+^ from the culture medium did not significantly affect the 4% or 20% stretch‐induced Ca^2+^ elevation (Figure 3E) or cell volume reduction (Figure 3F), nor did it induce cell damage under a 20% stretch (Figure S7A,B, Supporting Information). However, pretreatment with Thapsigargin to inhibit the endoplasmic reticulum (ER) Ca^2+^ ATPase, thereby depleting ER Ca^2+^ stores prior to stretch at 4% or 20%, effectively eliminated the increase in intracellular Ca^2+^ (Figure 3G) and the corresponding volume reduction (Figure 3H), and even resulted in cell damage under the normally non‐damaging 20% stretch (Figure S7C,D, Supporting Information). These findings demonstrate that ER Ca^2+^ release, rather than extracellular Ca^2+^ influx, is crucial in inducing cell shrinkage and mechanical adaptation.

Despite confirming the role of ER Ca^2+^ in mechanical adaptation, the specific mechanisms underlying the nonlinear elevation of intracellular Ca^2+^ levels remain elusive. State transitions in complex biological systems can be driven by positive feedback mechanisms, which amplify environmental signals and propel the system toward a new state beyond a critical threshold, exhibiting switch‐like nonlinear behavior.^[^ ^8^, ^34^, ^35^, ^36^ ^]^ Therefore, we hypothesized that the nonlinear elevation in intracellular Ca^2+^ level and the subsequent nonlinear adaptation emergence in response to stretch were mediated by the positive feedback mechanism. To verify this, we developed a mathematical model to describe the dynamics of stretch‐induced intracellular Ca^2+^ changes. This model delineates the changes in intracellular Ca^2+^ into four parts: force‐induced Ca^2+^ release, Ca^2+^‐induced positive feedback for Ca^2+^ release, Ca^2+^‐triggered Ca^2+^ reuptake, and leak Ca^2+^ influx. The process of positive feedback was modeled using the Hill equation, traditionally used to describe positive synergistic effects or feedback in biological processes.^[^ ^37^, ^38^ ^]^ The activation site and inactivation site of the receptors that mediated Ca^2+^ feedback were taken into consideration. The model parameters were then derived by fitting the model to experimental data of Fluo‐4 fluorescence intensity under various stretch amplitudes. The results demonstrated that the coefficient of determination (R^2^) was 0.96 (Figure 3I), indicating a strong correlation between the model and experimental data, and the Hill coefficient for Ca^2+^ at the activation sites was 2.06, greater than 1, suggesting the presence of positive feedback in the process of stretch‐induced Ca^2+^ nonlinear elevation. Additionally, the normalized experimental data of stretch amplitude‐cell volume (Figure 3J) and stretch amplitude‐cell damage ratio (Figure 3K) correlated strongly with normalized stretch amplitude‐ Ca^2+^ curves, with R^2^ values of 0.891 and 0.966, respectively, highlighting a strong association among Ca^2+^ levels, cell volume, and mechanical adaptation.

Furthermore, we investigated the specific regulator that drives Ca^2+^‐dependent positive feedback. Prior studies have demonstrated that calcium‐induced calcium release (CICR) acts as a positive feedback system, where elevated intracellular Ca^2+^ triggers further opening of calcium channels through binding to specific receptors in ER/SR (sarcoplasmic reticulum), leading to additional Ca^2+^ release.^[^ ^39^, ^40^ ^]^ Consequently, we hypothesized that specific receptors facilitating CICR might play a crucial role in mediating the positive feedback response to mechanical stretch. Increased intracellular Ca^2+^ activates inositol trisphosphate receptors (IP3R) and ryanodine receptors (RyR), mobilizing Ca^2+^ release from SR/ER stores.^[^ ^41^ ^]^ We then performed qPCR analysis to identify the involved Ca^2+^ receptors and discovered that expression of *RyR3* was upregulated in cells stretched at 4% (Figure S7E, Supporting Information). To further investigate RyR's role in this mechanism, we used the RyR inhibitor Dantrolene and observed that it suppressed the steep increase in intracellular Ca^2+^ induced by stretch (Figure 3L; Figure S6E, Supporting Information), hindered cell volume reduction under stretch (Figure 3M), and even caused cell damage at normally non‐damaging 20% stretch (Figure S7F,G, Supporting Information). Notably, we assessed the Ca^2+^ response in Dantrolene‐pretreated cells subjected to varying amplitudes of mechanical stretch and found intracellular Ca^2+^ levels closely matched the prediction of the mathematical model that excluded the positive feedback component, with an R^2^ value of 0.982 (Figure 3N), indicating that RyR‐mediated Ca^2+^ positive feedback is critical in nonlinear Ca^2+^ elevation under stretch. To validate the model's accuracy further, we introduced ionomycin into the culture medium to elevate intracellular Ca^2+^ in unstretched cells,^[^ ^42^ ^]^ which resulted in a nonlinear increase in intracellular Ca^2+^ over time (Figure 3O). Dantrolene treatment also blocked the ionomycin‐induced intracellular Ca^2+^ elevation (Figure 3O), confirming RyR's role in nonlinear Ca^2+^ elevation. Additionally, the initial and peak intracellular Ca^2+^ levels under ionomycin treatment closely followed the prediction of the mathematical model that omitted the “force‐induced Ca^2+^ release” component, with an R^2^ value of 0.893 (Figure 3P), demonstrating that stretch‐induced Ca^2+^ elevation triggers RyR‐mediated Ca^2+^ positive feedback. The agreement between model prediction and experimental data supports the validity of the mathematical model including Hill equation for describing intracellular Ca^2+^ dynamic in response to stretch. Collectively, these findings confirm that nonlinear cell shrinkage under mechanical stretch and the subsequent emergence of mechanical adaptation are driven by RyR‐mediated Ca^2+^ positive feedback.

### 2.4 Ca^2+^‐Dependent EVs Release‐Mediated Membrane Cholesterol Efflux Activates VRAC for Stretch‐Induced Cell Shrinkage

**Figure 4:**
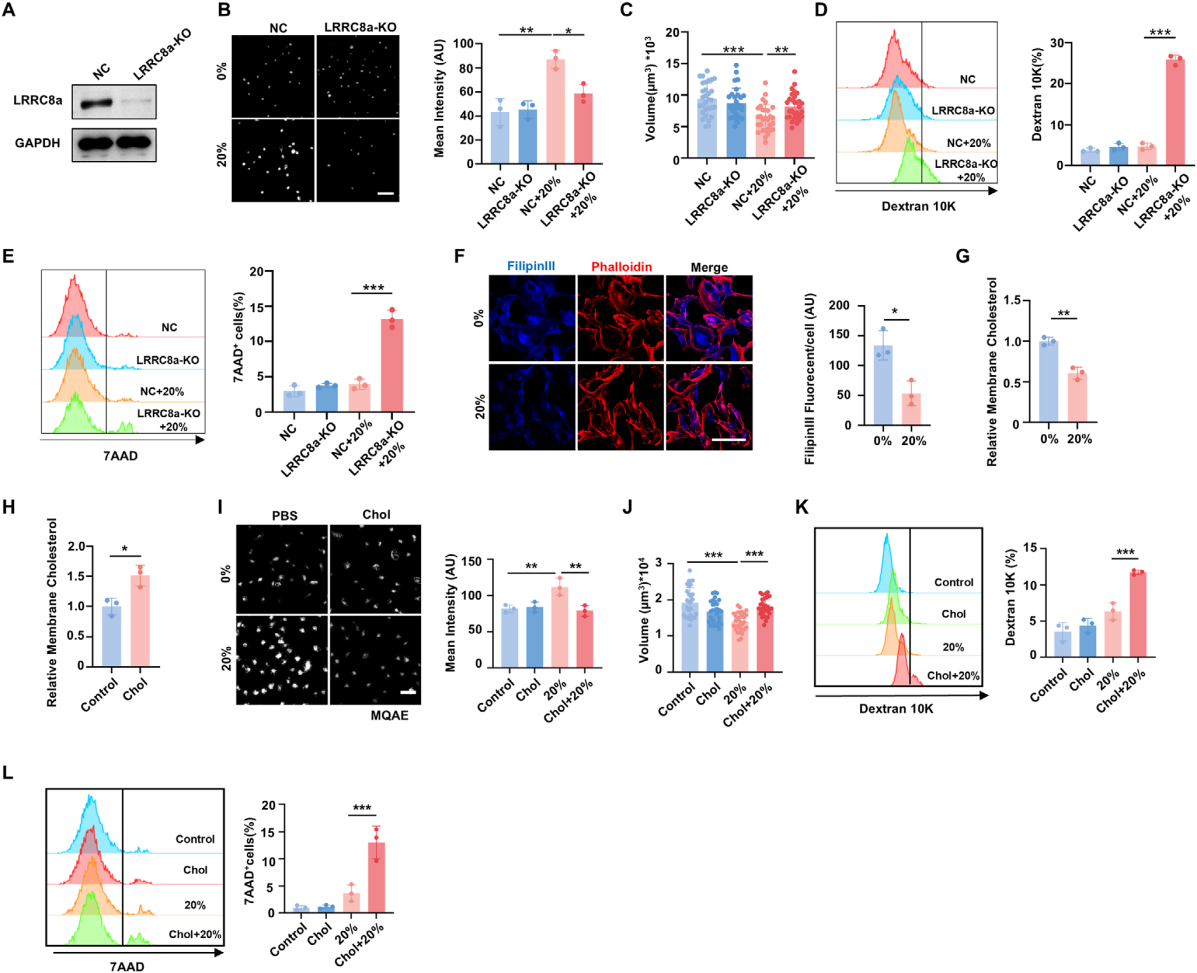
Stretch‐induced cell shrinkage is conducted by the activation of VRAC through membrane cholesterol efflux. A) Western Blot analysis confirmed the LRRC8a KO efficiency. B) Representative fluorescence images of Cl^−^ sensor MQAE detected by CLSM and quantitative fluorescence intensity. Scale bar, 20 µm. C) Cell volume analyzed by Imaris. n > 25 cells per group. D,E) Representative histograms of Dextran 10K‐FITC penetration (D) and 7AAD staining (E) and quantitative analysis. n  =  3 biologically independent samples. F) Representative fluorescence images of cells stained with FilipinIII, a fluorescent dye that binds specifically to cholesterol, and quantitative fluorescence intensity. Scale bar, 20 µm. n  =  3 biologically independent samples. G) Quantification of membrane cholesterol content by cholesterol oxidase‐mediated enzyme coupling reactions. H) Membrane cholesterol content in BEAS‐2b cells treated with water‐soluble cholesterol/methyl‐β‐cyclodextrin complex (Chol). n  =  3 biologically independent samples. I,J) BEAS‐2b cells pretreated with or without Chol were subjected to 20% stretch to evaluate the effects of cholesterol supplementation on subsequent investigations. (I) Representative fluorescence images and quantification of MQAE intensity. Scale bar, 20 µm. n  =  3 biologically independent samples. (J) Cell volume analyzed by Imaris. n > 25 cells from three independent experiments. K,L) Representative histograms and quantification analysis of Dextran 10K‐FITC penetration (K) and 7AAD staining (L) n  =  3 biologically independent samples. Data are presented as the mean ± s.d. Statistical significance was assessed by one‐way ANOVA with Tukey's post hoc test (A‐E, I‐L) and unpaired two‐tailed Student's t‐test (F‐H).

Given that intracellular Ca^2+^ elevation leads to cell shrinkage and the emergence of mechanical adaptation, we further explored the mechanism underlying cell volume regulation mediated by Ca^2+^. Mammalian cells regulate their volume by transporting ions and small molecule organic osmolytes through plasma membrane channels and transporters, creating an osmotic gradient that drives water flow in and out of the cells.^[^ ^30^, ^43^, ^44^, ^45^ ^]^ Since Cl^−^ was the main ion for cells to adjust cell volume,^[^ ^44^ ^]^ we first used the Cl^−^ sensor (fluorescent probe, N‐(6‐methoxyquinolyl) acetoethyl ester (MQAE)) to detect chloride channel activity. Meanwhile, the critical threshold to induce Cl^−^ reduction was identified between 3% and 4% stretch (Figure S8A, Supporting Information). A key chloride ion channel that mediates the reduction of cell volume is the volume‐regulated anion channel (VRAC),^[^ ^43^ ^]^ which is activated to reduce cell volume following cell swelling and also participates in physiological processes such as cell migration^[^ ^46^ ^]^ and T‐cell activation.^[^ ^47^ ^]^ However, it remains unknown whether VRAC participates in mechanical force‐induced cell volume reduction. Since LRRC8a is the essential subunit of VRAC, we generated LRRC8a knockout (KO) cells via CRISPR/Cas9 system to functionally inhibit VRAC. Western blot confirmed successful LRRC8a deletion (**Figure** **4A**). LRRC8a deficiency significantly attenuated the stretch‐induced Cl⁻ efflux (Figure 4B) and abolished the stretch‐induced cell volume reduction observed in negative control (NC) cells (Figure 4C). Furthermore, exposure to 20% stretch resulted in significantly higher damage to LRRC8a‐KO cells compared with NC cells (Figure 4D,E). Collectively, these findings indicated that VRAC is crucial for stretch‐induced cell volume decrease and the emergence of mechanical adaptation.

We then explored the upstream regulator of VRAC in response to stretch. Ion channel activity is proved to be modulated by the biophysical properties of plasma membrane.^[^ ^48^ ^]^ Given our observations of membrane folding and tension alterations upon stretch, we proposed that modulators of membrane properties, such as cholesterol, which is involved in the regulation of mechanosensitive ion channels,^[^ ^49^ ^]^ may regulate VRAC activation in response to mechanical stretch.^[^ ^50^, ^51^ ^]^ To assess cholesterol's role in VRAC activity upon stretch, we first quantified membrane cholesterol content. Filipin III staining showed a decrease in membrane cholesterol levels after a 4% or 20% stretch (Figure 4F; Figure S8B, Supporting Information), which was further confirmed through the Amplite Cholesterol Quantitation Kit (Figure 4G). Subsequently, we modulated membrane cholesterol levels to elucidate their impact on VRAC activation during stretch. Our findings revealed that supplementing membrane cholesterol with a water‐soluble cholesterol/MeβCD complex^[^ ^52^ ^]^ effectively increased cholesterol levels (Figure 4H) and significantly inhibited stretch‐induced cytoplasmic Cl^−^ efflux (Figure 4I), demonstrating VRAC activation inhibition. Notably, cholesterol supplementation not only prevented the typical volume reduction induced by stretch (Figure 4J) but also led to significant cell damage under a 20% stretch (Figure 4K,L). These data suggest that VRAC‐mediated stretch‐induced cell shrinkage is contingent upon membrane cholesterol reduction. Stretch‐induced plasma membrane cholesterol efflux and VRAC inhibition were also confirmed in alveolar epithelial cells, reinforcing the robustness of our experimental conclusions (Figure S9A–E, Supporting Information).

**Figure 5:**
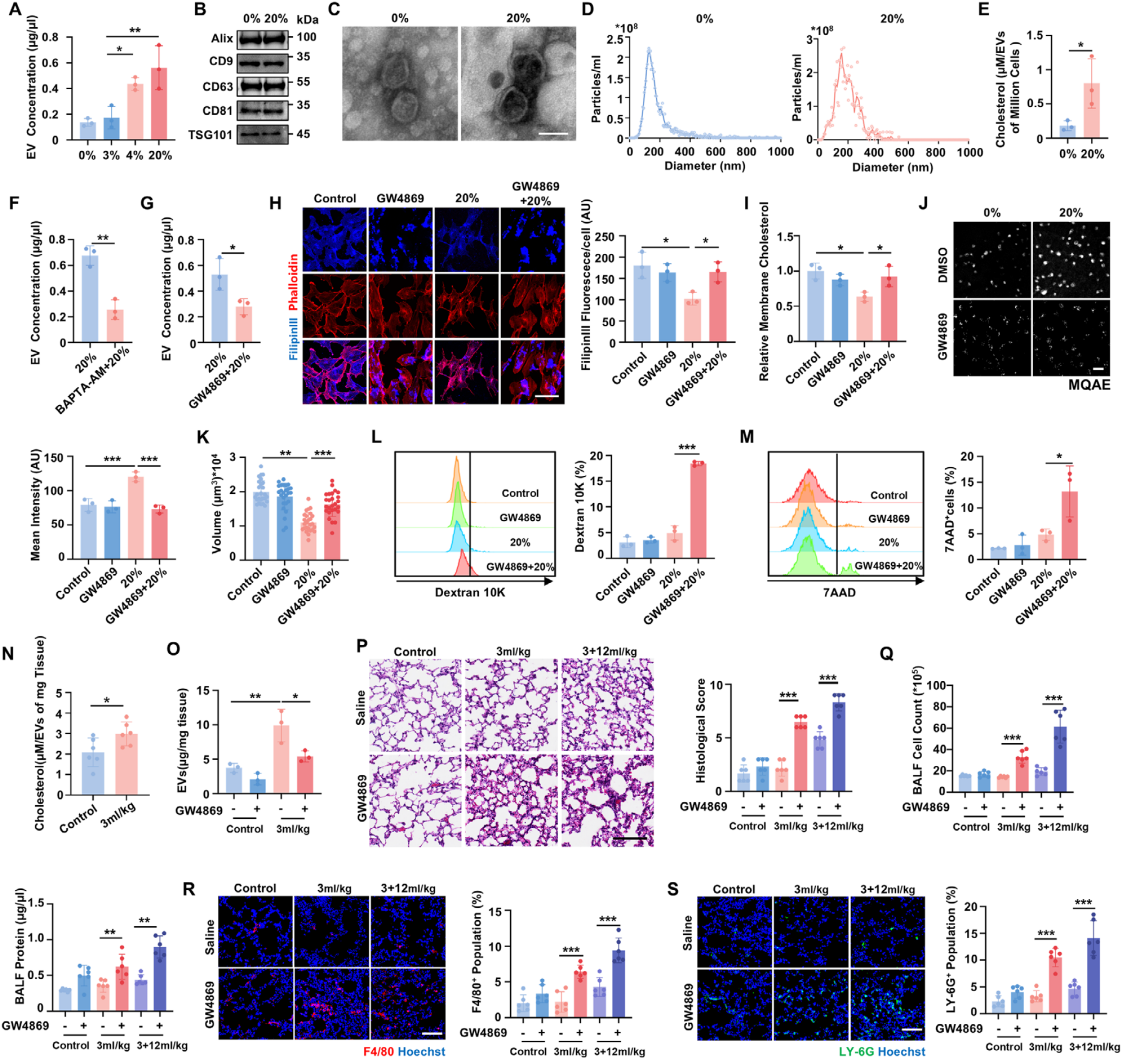
Stretch‐induced cell membrane cholesterol efflux was mediated by Ca^2+^‐dependent EVs release. A–E) EVs were isolated from the cell culture medium and characterized. A) The concentration of EVs released from an equivalent number of cells, was analyzed by BCA assay. n  =  3 biologically independent experiments. B) Western blot analysis of EVs markers Alix, CD9, CD63, CD81, and TSG101. C) Representative TEM images of EVs. Scale bar, 100 nm. n  =  3 biologically independent experiments. D) NTA analysis of EVs concentration and size distribution. E) Cholesterol content in EVs derived from the same number of cells. n  =  3 biologically independent experiments. F) The concentration of EVs released from an equivalent number of stretched cells with or without the pretreatment of BAPTA‐AM. n  =  3 biologically independent experiments. G) The concentration of EVs released from an equivalent number of stretched cells with or without the pretreatment of GW4869. n  =  3 biologically independent experiments. H–M) BEAS‐2b cells pretreated with or without GW4869 were subjected to 20% stretch to evaluate the effects of EVs release blockade on subsequent investigations. H) Representative fluorescence images of FilipinIII staining and quantitative fluorescence intensity. Scale bar, 20 µm. n  =  3 biologically independent experiments. I) Quantification of membrane cholesterol content. n  =  3 biologically independent experiments. J) Representative fluorescence images of MQAE and quantification of fluorescence intensity. Scale bar, 20 µm. n  =  3 biologically independent experiments. K) Cell volume analyzed by Imaris. n > 25 per group from three independent experiments. L,M) Representative histograms of Dextran 10K‐FITC penetration (L) and 7AAD staining (M) and quantitative analysis. n  =  3 biologically independent experiments. N–R) Mice were intratracheally injected with GW4869 and then subjected to ventilation, and lung tissues were collected for further analysis. (N) Cholesterol content in EVs derived from lung tissues. n  =  6 biologically independent samples. (O) The concentration of EVs derived from lung tissues after ventilation. n  =  3 biologically independent samples. (P) Representative images of H&E staining of lung sections and corresponding histological scores. Scale bar, 100 µm. n = 6 mice (Q) Cell counts and protein concentration in BALF. n  =  6 mice. (R) Representative fluorescence images of F4/80^+^ macrophage (red) in lung sections, with quantification of infiltrated cells. (S) Representative fluorescence images of LY‐6G^+^ neutrophils (green) in lung sections, with quantification of infiltrated cells. Scale bar, 100 µm. n  =  6 mice. Data are presented as the mean ± s.d. Statistical significance was assessed by one‐way ANOVA with Tukey's post hoc test (A, H‐J, L‐M, O‐S), Kruskal‐Wallis test (K, P) and unpaired two‐tailed Student's t‐test (E‐G, N).

Next, we elucidated the mechanism of stretch‐induced cholesterol efflux and cell membrane cholesterol reduction. The cellular vesicles are critical agents for cholesterol transport between intracellular compartments^[^ ^53^ ^]^ and extracellular vesicles (EVs) participate in extracellular cholesterol transport,^[^ ^54^ ^]^ potentially facilitating cholesterol efflux. Supporting prior findings that mechanical forces increase EVs release by cells,^[^ ^55^ ^]^ we observed an increase in EVs concentration in the condition medium of cells following a 4% or 20% stretch compared to control or cells treated with a 3% stretch, as determined by bicinchoninic acid (BCA) analysis (**Figure** **5**A). We then hypothesized that EVs released during stretch might also mediate membrane cholesterol efflux. The isolated EVs, were characterized by typical markers such as CD63, CD81, TSG101, and Alix (Figure 5B), and exhibited circular or elliptical morphology in transmission electron microscopy (TEM) images (Figure 5C). Nanoparticle tracking analysis (NTA) indicated that EVs released by stretched cells were larger in diameter than those from unstretched controls (Figure 5D), suggesting a distinct pathway for stretch‐induced EVs release. Notably, EVs from stretched cells contained higher cholesterol levels than those from controls (Figure 5E), indicating that mechanical stretch enhances the release of cholesterol‐rich EVs, contributing to membrane cholesterol reduction.

We further investigated whether intracellular Ca^2+^ elevation induced by stretch prompted EVs release, which in turn mediated membrane cholesterol reduction and VRAC activation. Initially, we used the calcium chelator BAPTA‐AM to inhibit intracellular Ca^2+^ before stretch and found that chelation attenuated EVs release (Figure 5F), underscoring the critical role of Ca^2+^ in EVs release. Additionally, we employed GW4869, commonly used to inhibit EVs release in vitro and in vivo,^[^ ^56^ ^]^ to examine the role of EVs release in cholesterol efflux and its downstream effects (Figure 5G). Blocking EVs release with GW4869 significantly curtailed membrane cholesterol efflux, as evidenced by Filipin III staining (Figure 5H) and cholesterol quantitative assays (Figure 5I). This blockade also inhibited VRAC activation in stretched cells, as indicated by MQAE mean intensity (Figure 5J). Furthermore, blocking EVs release inhibited the volume reduction (Figure 5K) and led to significant cell damage under a 20% stretch (Figure 5L,M). The process was also confirmed in alveolar epithelial cells (Figure S10A–D, Supporting Information). A consistent conclusion was reached when EVs release was inhibited by knocking down cytosolic Rab27a (Figure S11A,B, Supporting Information), which is involved in EVs release.^[^ ^57^, ^58^ ^]^ Rab27a knockdown in BEAS‐2b cells inhibited cholesterol efflux (Figure S11C,D, Supporting Information) and VRAC activation (Figure S11E, Supporting Information) induced by stretch, thereby suppressing the volume reduction (Figure S11F, Supporting Information) and increasing the percentage of damaged cells caused by stretch (Figure S11G,H, Supporting Information). These results demonstrate that Ca^2+^‐dependent EVs release mediates membrane cholesterol reduction and VRAC activation during stretch, establishing mechanical adaptation.

Then, we assessed the role of EVs release in mechanical adaptation against damaging ventilation in vivo. We isolated EVs from lung tissues of mice subjected to mechanical ventilation and found that EVs derived from ventilated lungs exhibited significantly elevated cholesterol content compared to that isolated from control animals under spontaneous breathing conditions (Figure 5N). Further, intratracheal injection of GW4869 was administered to inhibit EVs release in lung tissue (Figure 5O). We found that blocking EVs release compromised the protective effects generated by mild ventilation, rendering the mice unable to tolerate even non‐damaging mild ventilation at 3 ml kg^−1^, as evidenced by histological analysis (Figure 5P), alveolocapillary permeability (Figure 5Q), inflammatory cell infiltration (Figure 5R,S), and inflammatory factor secretion (Figure S11I, Supporting Information). Therefore, these findings demonstrate that during stretch, intracellular Ca^2+^ elevation‐induced EVs release carries cholesterol away from the cell membrane, thereby activating VRAC to achieve cell shrinkage and contribute to the emergence of mechanical adaptation.

### 2.5 Ca^2+^‐Dependent ANO6 Activation Contributes to EVs Release Upon Stretch

**Figure 6:**
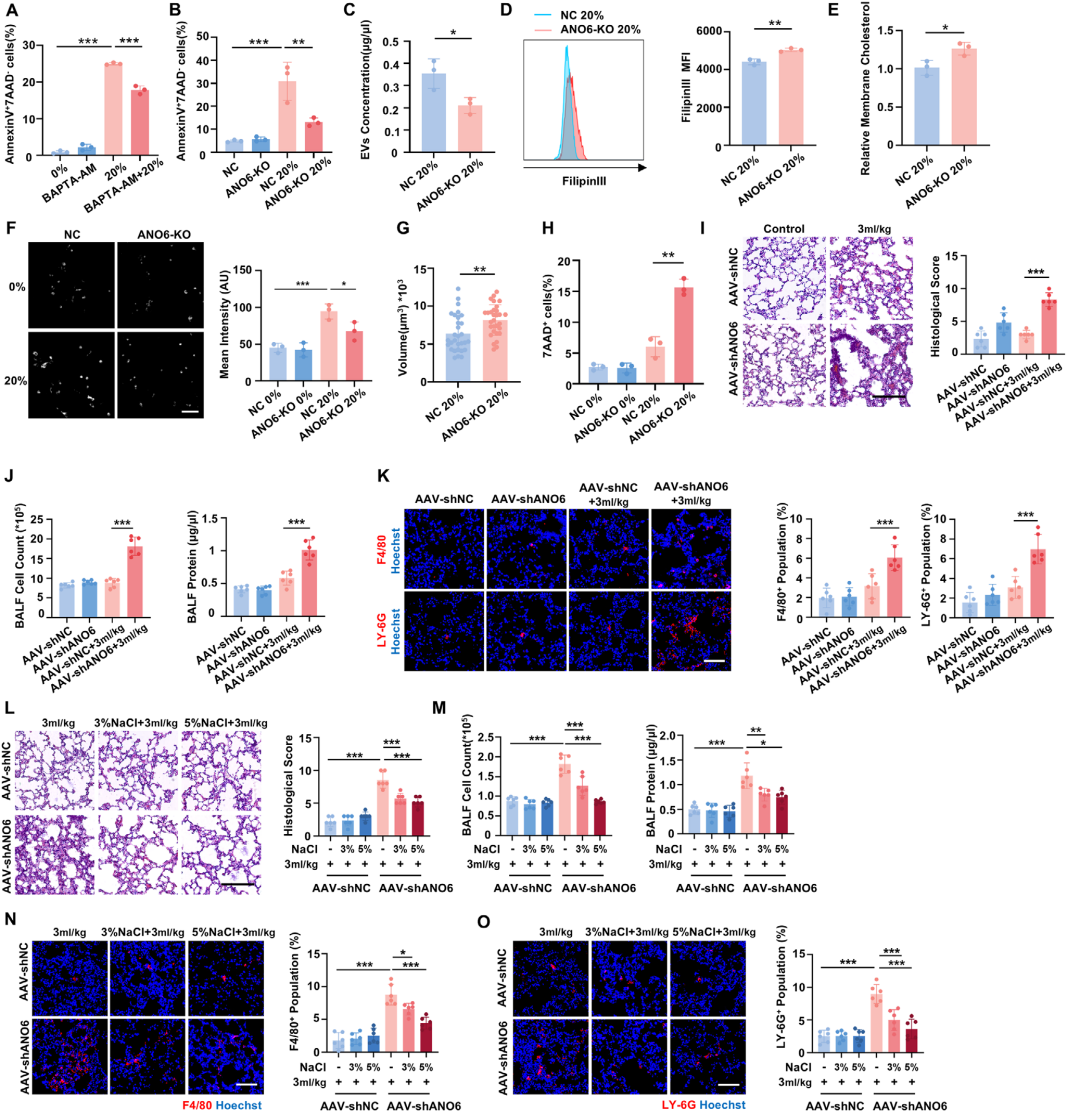
Ca^2+^‐dependent ANO6 activation contribute to EVs release upon stretch. A) Flow cytometry analysis of AnnexinV^+^7AAD^−^ cell percentage to assess membrane lipid scrambling in 20% stretched cells with or without pretreatment of BAPTA‐AM. n  =  3 biologically independent experiments. B) Flow cytometry analysis of AnnexinV⁺7AAD⁻ cell percentages in ANO6‐KO and NC cells following mechanical stretch. n  =  3 biologically independent experiments. C) The concentration of EVs released from an equivalent number of 20% stretched cells. n  =  3 biologically independent experiments. D) Representative histograms of Filipin III staining and MFI quantification analysis. E) Quantification of membrane cholesterol content by Amplite Cholesterol Quantitation Kit. n  =  3 biologically independent experiments. F) Representative fluorescence images of MQAE and quantification of fluorescence intensity. Scale bar, 20 µm. n  =  3 biologically independent experiments. G) Cell volume analyzed by Imaris. n > 25 per group from three independent experiments. H) Quantitative analysis of 7AAD^+^ cell percentage. n  =  3 biologically independent experiments. I–K) To knockdown ANO6 expression in lung tissue, mice were intratracheally injected with adeno‐associated virus (AAV) carrying shANO6‐ZsGreen (AAV‐shANO6) with shNC‐ZsGreen (AAV‐shNC) as negative control. After ventilation, lung tissues were collected for further analysis. (I) Representative images of H&E staining of lung sections and histological scores. Scale bar, 100 µm. n = 6 mice. (J) Cell counts and protein concentration in BALF. n  =  6 mice. (K) Representative fluorescence images of F4/80^+^ macrophages and LY‐6G^+^ neutrophils detected by CLSM and the percentage analysis. Scale bar, 100 µm. n = 6 mice. L–O) The protective effect of hyperosmotic nebulization pretreatment against VILI was evaluated in ANO6 knockdown mice. (L) Representative images of H&E staining of lung sections and histological scores. Scale bar, 100 µm. n = 6 mice. (M) Cell counts and protein concentration in BALF. N = 6 mice. (N‐O) Representative fluorescence images of F4/80^+^ macrophages (N) and LY‐6G^+^ neutrophils (O) in lung detected by CLSM, with quantification of infiltrated cells. Scale bar, 100 µm. n = 6 mice. Data are presented as the mean ± s.d. Statistical significance was assessed by one‐way ANOVA with Tukey's post hoc test (A‐B, F, H‐O) and unpaired two‐tailed Student's t‐test (C‐E, G).

Given that Ca^2+^‐dependent EVs release is essential for establishing cellular mechanical adaptation, we further investigated how Ca^2+^ promotes EVs release. Membrane phospholipid scrambling is an important trigger for EVs release,^[^ ^59^ ^]^ as it disrupts lateral membrane pressure, leading to membrane curvature changes and exocytosis.^[^ ^60^, ^61^ ^]^ Notably, we observed phosphatidylserine (PS) exposure, indicative of membrane lipid scrambling, at 4% and 20% stretch, detected by AnnexinV positive cell ratio (Figure S12A,B, Supporting Information). This lipid scrambling was inhibited by pretreatment with the calcium chelator BAPTA‐AM (**Figure** **6**A), demonstrating it is Ca^2+^ dependency. Although PS exposure is usually associated with apoptosis, the stretch‐induced PS exposure in this study was not accompanied by apoptosis, as evidenced by the absence of cleaved Caspase‐3 (Figure S12C, Supporting Information) and TUNEL staining (Figure S12D, Supporting Information), and was reversible 12 h post‐stretch (Figure S12E, Supporting Information). These findings suggest that PS exposure may serve as a distinctive marker of the cellular mechanical adaptive state.

We then sought to identify the Ca^2+^‐dependent regulator responsible for stretch‐induced lipid scrambling and EVs release. Anoctamin 6 (ANO6), also known as TMEM16F, is a Ca^2+^‐dependent lipid scramblase that mediates PS exposure in response to intracellular Ca^2+^ elevation.^[^ ^62^ ^]^ ANO6‐mediated PS exposure has been reported to regulate platelet activation^[^ ^63^ ^]^ and membrane repair through membrane blebs^[^ ^64^ ^]^ and EVs release.^[^ ^65^ ^]^ Importantly, ANO6 and other anoctamins also play a critical role in cellular volume regulation^[^ ^66^ ^]^ and volume‐regulated anion channel (VRAC) activation.^[^ ^67^, ^68^ ^]^ Consequently, we constructed ANO6‐KO cells through CRISPR/Cas9 system to elucidate the role of ANO6 in mediating cellular adaptation to mechanical forces (Figure S13A, Supporting Information). ANO6 deficiency significantly attenuated stretch‐induced PS exposure (Figure 6B), concurrently reducing EVs release (Figure 6C). Furthermore, ANO6 deficiency also inhibited stretch‐induced membrane cholesterol efflux (Figure 6D,E), impaired VRAC activation as indicated by MQAE fluorescence intensity (Figure 6F), and prevented cell volume reduction (Figure 6G). ANO6‐KO cells were also more susceptible to injury following 20% stretch compared to control (NC) cells, as indicated by an increased 7AAD^+^ cell ratio (Figure 6H). Similarly, the calcium‐dependent PS exposure and the ANO6‐mediated cellular mechanical adaptation were validated in BEAS‐2b (Figure S13B–K, Supporting Information).

We then investigated the protective role of ANO6 in lung injury induced by mechanical ventilation. To achieve ANO6 knockdown in lung tissue, we administered an adeno‐associated virus (AAV) vector expressing short hairpin RNA (shRNA) targeting ANO6 (AAV‐shANO6) via intratracheal injection. This knockdown significantly reduced ANO6 expression in the lungs, as confirmed by western blot and fluorescence images (Figure S13L,M, Supporting Information). Subsequent analysis revealed that ANO6 knockdown in the lung exacerbated lung injury, even with mild ventilation at 3 ml kg^−1^, as shown by increased histological scores (Figure 6I), elevated alveolocapillary permeability (Figure 6J), and enhanced inflammatory cell infiltration (Figure 6K) and inflammatory factor secretion (Figure S13N, Supporting Information). Importantly, nebulization with 3% or 5% hyperosmotic saline before mechanical ventilation markedly alleviated lung injury in ANO6 knockdown mice (Figure 6L–O; Figure S13O, Supporting Information). Taken together, these findings indicate that ANO6 activation in response to Ca^2+^ elevation under mechanical stretch promotes EVs release, cell shrinkage, and the establishment of mechanical adaptation.

Collectively, our findings demonstrate that stretch reaching a critical amplitude threshold induces Ca^2+^‐mediated positive feedback, leading to nonlinear Ca^2+^ elevation that activates ANO6, enhancing EVs release‐mediated membrane cholesterol efflux. The reduction in membrane cholesterol subsequently activates VRAC, promoting cell shrinkage and reducing membrane tension, ultimately establishing adaptation to mechanical stress. These results reveal a previously unknown decision‐making mechanism underlying cellular adaptation to mechanical stress.

## 3 Discussion

Adaptation, the ability of a system to adjust its activities to maintain essential functions in the face of errors, faults, or environmental perturbations, is a defining feature of complex systems^[^ ^69^ ^]^ such as ecological systems,^[^ ^70^ ^]^ biological systems^[^ ^1^ ^]^ and even individual cells.^[^ ^71^ ^]^ Mechanical forces play a crucial role in the physiological processes of living organisms. To avoid mechanical injury, organisms have evolved mechanisms to cope with mechanical stress. Evidence indicates that adaptive responses such as membrane reserve unfolding (e.g., ruffles or caveolae),^[^ ^7^ ^]^ nuclear softening,^[^ ^6^ ^]^ and actin cytoskeleton remodeling^[^ ^72^ ^]^ are key strategies for mechanical adaptation. However, it remains unclear whether these adaptations can evolve into stable states that confer cellular adaptation against subsequent mechanical challenges. Here, we uncover how cell states transform under mechanical forces, elucidating the features of cellular mechanical adaptive state and the underlying molecular mechanisms, thereby expanding our understanding of biological system responses to mechanical stress. Furthermore, understanding the fundamental principles that govern adaptation in biological systems may aid in developing treatments for injury or disease.

Importantly, our findings indicate that the emergence of adaptation is not directly proportional to the intensity of mechanical stress but instead develops in a nonlinear pattern in both in vivo and in vitro models. Nonlinearity is a fundamental characteristic inherent in systems across various scales, from microbial populations^[^ ^73^ ^]^ to global ecosystems,^[^ ^74^ ^]^ and is commonly observed in biological processes.^[^ ^13^, ^75^, ^76^, ^77^, ^78^ ^]^ Increasing evidence suggests that many regulatory elements responding to internal signals or external stimuli exhibit nonlinear, switch‐like behaviors. For instance, action potential conduction requires the membrane voltage to reach a critical threshold^[^ ^10^ ^]^ and full‐scale hair regeneration is triggered only when hair plucking reaches specific thresholds.^[^ ^13^ ^]^ Similarly, aging and changes in numerous aging‐related molecules also exhibit nonlinear dynamics,^[^ ^75^ ^]^ as do cell differentiation,^[^ ^77^ ^]^ immune response by lymphocytes,^[^ ^76^ ^]^ and acute exacerbation of chronic diseases.^[^ ^78^ ^]^ Interestingly, we observed that cellular adaptation to other stimuli, such as heat and hypo‐osmotic stress, also emerges in a nonlinear manner (Figure S4, Supporting Information). Heat adaptation may be mediated by the accumulation of heat shock proteins to maintain correct protein folding,^[^ ^79^ ^]^ while adaptation to hypo‐osmotic stress may be facilitated by the dissolution of biomolecular condensates to capture free water.^[^ ^80^ ^]^ Although distinct regulatory mechanisms likely underlie different forms of adaptation, these observations suggest that the nonlinear emergence of adaptation is a general feature across various types of environmental stress. We propose that this nonlinearity enables organisms to filter environmental noise, ensuring that responses are triggered only by sufficiently strong signals.

Uncovering the fundamental principles of adaptation facilitates the translation of the concept of mild pre‐stimulation‐induced adaptation into clinical practice. The existence of adaptation enables the prevention of potential damage through intrinsic protective mechanisms activated by targeted pre‐stimulation. For instance, ischemic preconditioning has been shown to provide cardioprotection, alleviating myocardial infarction in patients.^[^ ^81^ ^]^ Additionally, our findings that hyperosmotic saline nebulization mitigates mechanical ventilation injury suggest that signaling pathways involved in adaptive regulation could serve as molecular targets for pharmacological intervention. The application of pre‐stimulation‐induced adaptive responses can also provide a theoretical framework for reducing the potential damage caused by mechanical forces associated clinical treatments such as orthodontics and distraction osteogenesis. Notably, the nonlinear emergence pattern of adaptation offers a theoretical foundation for enhancing the precision of disease prevention and treatment strategies. By leveraging this nonlinear characteristic, treatment strategies can be tailored with precise intensity to avoid ineffective or overly aggressive interventions, thereby achieving optimal therapeutic outcomes.

Regarding the mechanisms underlying nonlinear adaptation, we found that Ca^2+^ signaling amplification, driven by a positive feedback mechanism mediated by RyR, is a key determinant in the nonlinear emergence of mechanical adaptation. Ca^2+^ is known to transmit acute signals in processes such as inflammation, wound healing,^[^ ^82^ ^]^ and stress responses.^[^ ^83^ ^]^ Recent studies indicate that Ca^2+^ signaling involves actin resetting and changes in cellular morphology,^[^ ^84^ ^]^ similar to the Ca^2+^‐mediated cell shrinkage observed in this study. Guided by a mathematical model of intracellular Ca^2+^ dynamics, we identified a positive feedback loop in Ca^2+^ signaling. Feedback regulation is a fundamental mechanism in living systems.^[^ ^36^ ^]^ Unlike negative feedback, which typically maintains system stability, positive feedback can amplify signals and drives the system into a new equilibrium state by breaking the existing balance.^[^ ^34^ ^]^ For example, in the coagulation process, the positive feedback between platelet activation and coagulation factors cascade accelerates blood clot formation.^[^ ^85^ ^]^ During parturition, the positive feedback between uterine contractions and the release of oxytocin enhances the contractions until a baby is born.^[^ ^86^ ^]^ In particular, the Ca^2+^ feedback process is also involved in muscle contraction.^[^ ^87^ ^]^ Positive feedback mechanisms allow cells to detect stress rapidly and transition into an adaptive state in a nonlinear manner, enabling an immediate response to environmental challenges.

The cellular adaptation induced by a nonlinear positive feedback process underscores the inherent complexity within biological systems. Using mechanical stimulation as a model, this study reveals foundational characteristics of complex systems and provides insights into how organisms respond and adapt to environmental perturbations. While our study focused on the emergence of adaptation, the process of adaptation loss was not thoroughly examined. Previous studies suggest that adaptation loss can also follow a nonlinear trajectory. For example, forest ecosystems show a nonlinear decline in resilience/adaptation against natural and anthropogenic perturbations.^[^ ^70^ ^]^ Additionally, recent studies in ecology^[^ ^70^ ^]^ and systems biology^[^ ^78^, ^88^ ^]^ indicate that slowed recovery following perturbations signals that complex biological systems are approaching a critical point of resilience loss, serving as an early indicator for predicting critical thresholds. Therefore, continued in‐depth research is necessary to understand the critical thresholds associated with both the emergence and loss of resilience/adaptation.

## 4 Experimental Section

### Cell Culture

Human bronchial epithelial cells (BEAS‐2b) (CRL‐3588, ATCC) were cultured in high glucose Dulbecco's Modified Eagle's medium (DMEM) supplemented with 10% fetal bovine serum and 1% penicillin‐streptomycin. Primary mouse type II alveolar epithelial cells (CP‐M003, Procell) were cultured in a specialized medium (CM‐M003, Procell). To promote cell adhesion, culture dishes for the primary mouse type II alveolar epithelial cells were coated with collagen type I (rat tail, Corning, NY, USA). MLE‐12 (CRL‐2110, ATCC) cell line were cultured in DMEM/F12 supplemented with 10% fetal bovine serum and 1% penicillin‐streptomycin. lymphoblastoid Raji cells (CL‐0189, Procell) or 293T cells (CL‐0005, Procell) were cultured in RPMI or DMEM, respectively, with 10% fetal bovine serum and 1% penicillin‐streptomycin. All cells were kept at 37 °C in a 5% CO_2_ atmosphere and passaged every 2–3 days using trypsin (Thermo). All cell lines were tested and found negative for mycoplasma contamination.

### Mechanical Stretching

BEAS‐2b cells, mouse type II alveolar epithelial cells, or MLE‐12 cells were cultured on Polydimethylsiloxane (PDMS) membranes (Transformer, TAIHOYA Corp.) precoated with collagen type I. For each experiment, 150000 cells per elastomer (10 cm^2^) were seeded and allowed to attach for 12 h prior to initiating experimental procedures. Cells were exposed to cyclic uniaxial stretch using ATMS Dynamic Culture System (Transformer, TAIHOYA Corp.) at the mentioned amplitudes and a frequency of 0.24 Hz.^[^ ^89^ ^]^ The unstretched cells were cultured under the same conditions without stretch. All stretch procedures were performed in a humidified atmosphere at 37 °C and 5% CO_2_.

### Cell Hypo‐Osmotic Stimulation

BEAS‐2b, Raji, and 293T cells were seeded on plates and incubated for 12 h respectively. Then, cells were digested with trypsin and placed in mild hypo‐osmotic solutions of varying concentrations, prepared with MilliQ water. After 10 min, cells were centrifuged and resuspended in a damaging hypo‐osmotic solution. They were subsequently stained with PI (40711ES10, YeaSen) and analyzed using a flow cytometer (Beckman Coulter).

### Cell Heat Stimulation

BEAS‐2b, Raji, and 293T cells were seeded·on plates and incubated for 12 h. Then, cells were trypsinized and resuspended in PBS, and subjected to different temperatures for 20 min, with temperature controlled by a precision water bath. Following the mild heat treatment, cells were transferred to an incubator at 37 °C to restore baseline temperature. Subsequently, the cells were exposed to damaging heat stress at a higher temperature for 3 min, stained with Dextran 10K (HY‐128868, MCE), and analyzed using a flow cytometer (Beckman Coulter).

### Animal Experiments

All animal experiments were approved by the Animal Care Committee of the Fourth Military Medical University (2023‐kq‐050). Wild‐type male and female 6‐8‐week‐old C57BL/6J mice were obtained from the Laboratory Animal Research Center of the Fourth Military Medical University. All mice were housed in a 12 h dark‐light cycle and had free access to food pellets and tap water. Mice were anesthetized by an intraperitoneal injection of pentobarbitone sodium (40 mg kg^−1^) The tracheal intubation was performed with a 20G catheter, and the mice were connected to a small animal mechanical ventilator (DW‐3000H Anhui Zhenhua Biological Instrument Equipment Co., Ltd). Body temperature was maintained by a gauze pad to cover the mice. The mice were ventilated for 4 h with the tidal volume of 12 ml kg^−1[^ ^90^ ^]^ and a respiratory rate of 100 breaths per minute, with or without 15 min pretreatment of mild low tidal volume ventilation. In the sham group, mice maintained spontaneous breathing following intubation. The mice in the nebulization group were nebulized with 3% or 5% hyperosmotic saline for 15 min using a nebulizer before mechanical ventilation.

To identify whether EVs release could attenuate lung injury caused by damaging ventilation in vivo, mice received an intratracheal injection with GW4869 at a dose of 2.5 µg g^−1^, administered 12 h before ventilation. GW4869 was initially dissolved in dimethylsulfoxide to a stock solution of 10 mg ml^−1^, and the working solution was further diluted in saline before use. Mice in the control group received equivalent injections of saline. After ventilation, mice were administered a lethal overdose of anesthetic, and BALF were collected by instilling 600 µl of PBS into the lungs twice and withdrawing it. BALF was then centrifuged for 10 min at 1000 *g* to obtain cells for counting, and the supernatant was collected for protein concentration analysis and ELISA. Lung tissues were harvested and prepared for histological evaluation, immunofluorescence staining, and MPO activity assays.

### Cell Damage Analysis

To evaluate cell damage, the penetration of Dextran 10K‐FITC (MW = 10000) and the 7AAD^+^ cell ratio were measured. Briefly, cells were collected, washed in PBS and centrifuged, Dextran 10K (5 µl 10 mg ml^−1^, HY‐128868, MCE) or 7AAD (5 µl, 559 763, BD) were added in 500 µl cell suspension and incubated for 10 min. The penetration of Dextran 10 K and 7AAD^+^ cell ratio was detected with CytoFLEX flow cytometer (Beckman Coulter).

### Cell Volume Measurement

Cells for volume measurement were transduced with Zoanthus sp. green fluorescent protein (ZsGreen) (Hanbio Tech, Shanghai, China). ZsGreen, which distributes uniformly throughout the cell body, was used as a fluorescent marker for detecting cell volume. Confocal stacks (20–40 steps, 1 µm step size) capturing the entire ZsGreen signal were acquired with CLSM (Nikon). Cells were randomly picked for analysis. 3D reconstruction of confocal stacks and volume calculation were performed using Imaris 9.01 software. Cell volume discrepancy was evaluated by forward scatter (FSC) on flow cytometry.

### Histological Evaluation

Lung tissue samples were fixed in 4% paraformaldehyde (PFA) for 24 h at 4 °C, embedded in paraffin, sectioned into 4‐µm sections (Leica RM2235 manual rotary microtome), The sections were prepared for hematoxylin and eosin staining and evaluated by histological index. The degree of lung injury was determined by histological scoring analysis. The assessment was expressed as the sum of the individual score grades of 0 (normal), 1 (mild injury), 2 (moderate injury), 3 (severe injury), and 4 (maximum injury) for each of the following four categories including inflammation infiltration, alveolar wall thickening, microhemorrhages, alveolar hyperinflation. The evaluators were blinded to group identity.

### Immunofluorescence Staining

Lung tissues samples were fixed in 4% PFA for 24 h and washed with PBS, immersed in 30% sucrose solution at 4 °C for 24 h and then embedded in optimum cutting temperature compound (Tissue OCT freeze Medium). The tissues were sectioned in 10‐µm sections. After permeabilization with 0.1% Triton X‐100 (T8787, Sigma), cryosections were blocked with goat serum (Boster) for 1 h at room temperature. Cryosections were then incubated overnight at 4 °C with the following primary antibodies: anti‐F4/80 antibody (1:200, ab6640, Abcam), anti‐LY‐6G antibody (1:200, sc‐53515, Santa Cruze), anti‐ANO6 antibody (1:200, 20784‐1‐AP, Proteintech). After washing with PBS, sections were incubated with Alexa Fluor 488/594‐conjugated secondary antibodies (1:200, YeaSen) for 1 h at room temperature. Nuclear staining was performed by incubating with Hoechst 33 342 (Thermo) for 15 min. Immunofluorescence images were captured by CLSM (Nikon) and analyzed by ImageJ 1.53 software (Fiji).

### Intracellular Ca^2+^ Measurement

To analyze the intracellular Ca^2+^ levels, cells were loaded with the cell‐permeant Ca^2+^ indicator Fluo‐4 AM (40704ES50, YeaSen) following the manufacturer's protocol. Briefly, after rinsed with calcium‐free Hank's balanced salt solution (60148ES76, YeaSen), cells were incubated in Fluo‐4 AM at a final concentration of 2 µm at 37 °C for 30 min. Then washed with HBSS and incubated for an additional 20 min. The green fluorescence of Fluo‐4 was captured by CLSM (Nikon), the fluorescence intensity of Fluo‐4 was analyzed by Imagej 1.53 software.

### EVs Isolation and Characterization

Cell supernatants were centrifuged at 800 *g* for 5 min to remove cells and cell debris. Then, the supernatant was ultracentrifuged at 150000 *g* for 70 min at 4 °C, washed with PBS, and purified by a second ultracentrifugation at 150000 *g* for 70 min. The concentration of EVs was measured by a BCA kit (Beyotime), and the size of EVs was examined using NTA with NanoSight NS300 (Malvern). The EVs derived from pulmonary tissue were isolated as a previous study reported.^[^ ^91^ ^]^ Briefly, fresh lung tissues were dissected and rinsed with ice‐cold PBS three times. Then, the tissues were cut into small pieces (2 mm × 2 mm × 2 mm) and incubated with collagenase I for 30 min at 37 °C. Filtration and low‐force centrifugation (300 *g* for 10 min, followed by 2000 *g* for 20 min at 4 °C) were used to remove cells and debris. The resulting supernatant was ultracentrifuged at 118000 *g* for 2.5 h at 4 °C to collect the EVs.

### Transmission Electron Microscopy

For EVs observation, a drop of suspension containing EVs was applied to a 200‐mesh carbon‐stabilized copper grid. EVs were allowed to settle for 3 min before the excess suspension was wicked off. Next, the grid was stained with 2% phosphotungstic acid hydrate for 30 s followed by washing it with distilled water three times. The excess solution was wicked off, and the grid was allowed to air‐dry before observation. For cell observation, cells were scraped and centrifuged, then fixed with 2% glutaraldehyde for 12 h. The samples were postfixed in 1% osmium tetroxide, rinsed with PBS, and dehydrated in gradient ethanol, and the samples were embedded in acetone and embedding agent mixture and then solidified in the oven at 60 °C for 48 h. 90 nm ultrathin sections were prepared and stained with uranyl acetate and lead citrate. Images were taken with the transmission electron microscope (TECNAI Spirit, FEI).

### Scanning Electron Microscopy

Cells on PDMS were fixed with 2% phosphotungstic acid and subjected to a graded ethanol dehydration series at concentrations of 30%, 50%, 70%, 80%, 90%, 95%, and 100% for 15 min each, followed by a 15 min treatment with isoamyl acetate. Samples were then dried using a critical point dryer (K850, Quorum). Subsequently, the specimens were mounted onto metallic stubs using carbon stickers and sputter‐coated with gold for 30 s. Imaging was performed using a scanning electron microscope (Hitachi).

### FilipinIII Staining

Cells on PDMS were fixed in 4% PFA. After washing with PBS, cells were then stained with FilipinIII (50 µg ml^−1^, SAE0087, Sigma) for 1 h at room temperature. The fluorescence images were obtained by CLSM (Nikon), the fluorescence histograms were obtained by flow cytometry (Beckman Coulter).

### Quantification of Membrane Cholesterol

Cells were collected using a cell scraper, rinsed with PBS, and centrifuged to obtain cell precipitate. The plasma membrane was then extracted using Minute Plasma Membrane Protein Isolation and Cell Fractionation Kit (SM‐005, Invent biotechnologies). For lipid extraction, the isolated cell membrane was added to methanol/chloroform mixture (1:2, v/v). After centrifuging at 8,000 rpm for 10 min, the organic phase containing cholesterol was collected, and the solvent was evaporated under vacuum. Cholesterol in each sample was quantified using the Amplite cholesterol quantitation kit (40 006, AAT Bioquest) according to the manufacturer's protocol. Plasma membrane cholesterol content was also quantified by subtracting intracellular cholesterol levels from total whole‐cell cholesterol measurements. Briefly, cells were treated with cholesterol oxidase (2 U ml^−1^ in PBS) to oxidize the plasma membrane, and then the intracellular cholesterol was detected. The plasma membrane cholesterol level was calculated by subtracting the intracellular cholesterol level from the whole cellular cholesterol level.

### Modulation of Cholesterol Levels in the Plasma Membrane

To supplement the cell plasma membrane with cholesterol, BEAS‐2b cells were treated with water‐soluble cholesterol/MeβCD complex (5 mm) in a complete medium at 37 °C for 30 min, and then washed with PBS twice.

### Phosphatidylserine Exposure Analysis

The Phosphatidylserine exposure ratio of BEAS‐2b cells was evaluated by Annexin V‐PE/7AAD apoptosis detection kit (#559 763, BD Bioscience) according to the manufactory's protocol. Briefly, cells were harvested using trypsin without EDTA and then washed with cold PBS and resuspended in 100 µl of binding buffer. Annexin V (5 µl) and 7AAD (5 µl) were added to cell suspension and incubated for 15 min. After incubation, 400 µl of binding buffer was added, and the samples were analyzed using flow cytometer. Besides, cells were also stained with AnnexinV‐FITC and fluorescence images were acquired through CLSM.

### TUNEL Staining

TUNEL detection followed the manufacturer's protocol. Briefly, cells were fixed in 4% PFA, and then stained with TUNEL working solution (C1086, Beyotime) for 60 min at 37 °C. After washing in PBS, fluorescence images were obtained by CLSM.

### Intracellular Cl^−^ Measurement

Intracellular Cl^−^ concentration was measured using MQAE (HY‐D0090, MCE). Briefly, cells were incubated with 5 mm MQAE in Krebs‐Hepes solution for 30 min at 37 °C. After washing with PBS, fluorescence images of MQAE were acquired at an excitation wavelength of 350 nm by CLSM.

### RNA Extraction and Quantitative Real‐Time PCR

Total RNA was isolated using TRIzol Reagent (MI00617, Mishu Shengwu) and quantified by Nanodrop (Thermo Fisher Scientific). Total RNA (1 µg) was converted into cDNA with the Reverse Transcription Kit (RR037A, Takara). Quantitative RT‐PCR analysis was performed on the CFX 96 Touch (BIO‐RAD) with SYBR Green Master Mix (RR820B, Takara). *GAPDH* was used as a normalization control. Relative expression of genes was calculated by the −2ΔΔCt method. Results were plotted using the GraphPad Prism 9. software (GraphPad Software).

The primers sequenced were listed below: *RyR1* (forward: 5′‐AAGCAGTCACCACAGGCGAGA‐3′; reverse: 5′‐CGGTGCCCATTGAAGACATAG‐3′), *RyR2* (forward: 5′‐TGGATGATGGCATAAGTTTGTCG‐3′; reverse: 5′‐AGGGCTCGTAGTCTGTTCTGTTT‐3′), *RyR3* (forward: 5′‐CTTCCTAACAGCAGAGCCCACA‐3′; reverse: 5′‐AGCAGCTTACCACGTCATCCG‐3′), *ITPR1* (forward: 5′‐TTAAGCTATGTCCCATGAACCG‐3′; reverse: 5′‐TTGTTGAGTAGCACTGCGTCTG‐3′), *ITPR2* (forward: 5′‐ACCCAGGGTGTAAAGAGGTGAA‐3′; reverse: 5′‐GAAGTAGCTGATTGGCGCAAG‐3′), *ITPR3* (forward: 5′‐ACAGAAGCAAGTTTGAGGAGAATGA‐3′; reverse: 5′‐CAGGTAGGATATGCGGTAATCCA‐3′), *GAPDH* (Forward: 5′‐TGTGTCCGTCGTGGATCTGA‐3′; Reverse: 5′‐TTGCTGTTGAAGTCGCAGGAG‐3′).

### Chemical Treatments

For pretreatment, 1% PEG300 (HY‐Y0873, MCE), Sorbitol (40 mm, HY‐B0400, MCE), BAPTA‐AM (10 µm, A1076, Sigma‐Aldrich), Thapsigargin (1 µM, HY‐13433, MCE) were added into the culture medium 15 min prior to mechanical stretch. Dantrolene (20 µm, HY‐12542, MCE) was added into the culture medium 60 min prior to mechanical stretch. Ionomycin (1 µm, 50401ES03, YeaSen) was added at the start of living imaging. GW4869 (10 µm, HY‐19363, MCE) was added into the culture medium 8 h prior to mechanical stretch.

### Transfection

BEAS‐2b cells were transduced with lentiviral vectors expressing shRNA targeting ANO6 (designated sh‐ANO6; Hanbio Tech) or Rab27a (designated sh‐Rab27a; Shanghai JiKai Gene). An identical lentiviral vector expressing a non‐specific shRNA served as the non‐targeting control (designated sh‐NC). Transduction was performed at a multiplicity of infection (MOI) of 10 in the presence of polybrene (4 µg ml^−1^). MLE‐12 cells were transduced with lentiviral vectors encoding sgRNA targeting LRRC8a (designated LRRC8a‐KO, Shanghai JiKai Gene) or ANO6 (designated ANO6‐KO; Shanghai JiKai Gene) to knockout target protein. Transduction was performed at a multiplicity of infection (MOI) of 50 in the presence of polybrene (4 µg ml^−1^). Transduced cells were selected using puromycin (1 µg mL^−1^) and monoclonal isolation. For animal experiments, an intratracheal injection of ANO6 adeno‐associated virus serotype 9 (AAV9) at a dose of 10^11^v.g was administered four weeks before mechanical ventilation in order to knockdown ANO6 expression in pulmonary tissue.

### ELISA

Enzyme‐linked immunosorbent assays (ELISA) were performed to assess secreted cytokine levels in BALF. IL‐1β (EMC001b.96, NeoBioscience), IL‐6 (EMC004.96, NeoBioscience), and TNF‐α (bsk12073, Bioss Antibodies) ELISA kits were used according to manufacturer's protocol.

### Membrane Tension Detection

Membrane stress sensor (MSS, AGM92262, Aogma)^[^ ^31^ ^]^ was used for membrane tension detection based on the fluorescence resonance energy transfer (FRET) technique, which comprising an elastic spider silk protein inserted between ECFP and YPet, and two anchoring proteins linked with lipid molecules in lipid raft and nonlipid raft regions. A higher FRET ratio indicates a lower membrane tension. Transfections were performed using Lipofectamine 2000 (Invitrogen) according to the manufacturer's instructions. After 72 h, transfected cells were subjected to stretch. FRET experiments were carried out using CLSM according to the manufacturer's instructions, and the FRET ratio images were formed by calculating YPet/ECFP.

### Mathematic Model

According to the biological mechanisms as mentioned in Results section, the mathematical model was developed to describe the dynamics of cytosolic Ca^2^⁺ concentration ([Ca^2 +^]):

(1) $$\begin{array}{ccc} \frac{d\left[Ca^{2+}\right]}{dt} & = & V_{\max}\frac{{\left[Ca^{2+}\right]}^{n_{A}}}{{\left[Ca^{2+}\right]}^{n_{A}}+K_{A}^{n_{A}}}\left(1-\frac{{\left[Ca^{2+}\right]}^{n_{I}}}{{\left[Ca^{2+}\right]}^{n_{I}}+K_{I}^{n_{I}}}\right)\\ & & +\;k_{F}F-k_{\mathrm{Ca}}\left[Ca^{2+}\right]+C \end{array}$$

where the variables [Ca^2 +^] and *F* represent are the Ca^2^^+^ concentration and the amplitude of stretch applied to the cell, respectively. The first term in Equation (1) employs Hill functions to describe the CICR, where *V_max_* represents the maximum release rate of Ca^2+^, *K_A_*, and *K_I_* are the dissociation constants for activation and inactivation, respectively, and *n_A_* and *n_I_* are the corresponding Hill coefficients for Ca^2^⁺ binding at the activation and inactivation sites.^[^ ^92^ ^]^ The remaining parameters are as follows: *k*_F_ is the force‐sensitive Ca^2+^ release coefficient, reflecting the sensitivity of the cell to force stimulation; *k*_Ca_ is the Ca^2+^ efflux rate constant, indicating the efficiency of Ca^2+^ efflux under elevated [Ca^2 +^]; and *C* represents the basal Ca^2+^ influx rate.

Through experimental measurements, steady‐state intracellular Ca^2^⁺ concentration data was obtained under various stretch force. At steady state, the rate of change of Ca^2+^ concentration approaches zero, expressed as $\frac{d[Ca^{2+}]}{dt}=0$, where $\frac{d[Ca^{2+}]}{dt}$ is defined by Equation (1). To estimate the parameters, a least‐squares fitting procedure was applied to the effective equations under the steady‐state assumption using the experimental data. The quantitative values of the parameters were as follows: *V*_max_ = 546.32, *K*_A_ = 2372.55, *K*_I_ = 1865.59, *k*_Ca_ = 0.06, *k*_F_ = 56.22, *C* = 11.41, *n*_A_ = 2.06, *n*_I_ = 1.61. The coefficient of determination, R^2^ was calculated to evaluate the degree of fit.

The intracellular Ca^2+^ dynamics under inhibition of RyR activation was described by the following mathematical equation:

(2) $$\frac{d\left[Ca^{2+}\right]}{dt}=k_{F}F-k_{\mathrm{Ca}}\left[Ca^{2+}\right]+C$$

The intracellular Ca^2+^ dynamics under ionomycin treatment was described by the following mathematical equation:

(3) $$\begin{array}{c} \frac{d\left[Ca^{2+}\right]}{dt}=V_{\max}\frac{{\left[Ca^{2+}\right]}^{n_{A}}}{{\left[Ca^{2+}\right]}^{n_{A}}+K_{A}^{n_{A}}}\left(1-\frac{{\left[Ca^{2+}\right]}^{n_{I}}}{{\left[Ca^{2+}\right]}^{n_{I}}+K_{I}^{n_{I}}}\right)-k_{\mathrm{Ca}}\left[Ca^{2+}\right]+C \end{array}$$

While the theoretical units for each term in Equations (1)–(3) are mol L^−1^⋅sec, the experimental data utilizes Ca^2+^ fluorescence intensity as a proxy for intracellular Ca^2+^ levels. Although a conversion factor could theoretically be applied to transform the intensity units into absolute concentrations (mol L^−1^), this would only result in a uniform scaling of the data and would not alter the conclusions regarding the relationship between stretch, Ca^2+^ dynamics, and cellular behavior. This study focuses on the relative changes in Ca^2+^ levels, and these relationships remain consistent regardless of the Ca^2+^ measurement methods used.

Furthermore, the cell volume and mechanical adaptation were influenced by Ca^2+^ concentration

(4) $$\begin{array}{c} \left[Ca^{2+}\right]\to \left[V\right]\\ \left[Ca^{2+}\right]\to Adaptation \end{array}$$

Normalized of cell volume (min = ‐0.16, max = 1.04) and cell damage (min = ‐0.05, max = 1.04) was done using min‐max normalization. The scaled experimental data were then compared with the predictions from the normalized model (Figure 3J–K).^[^ ^93^ ^]^

### Statistical Analysis

Data were presented as mean ± s.d and were analyzed using SPSS v.19.0 software. For all tests, differences of P < 0.05 were considered indicative of significance. Data normality and equal variances were assessed before comparisons. For normally distributed data with equal variances, two samples were compared using unpaired two‐tailed Student's t‐tests and multiple samples were compared using one‐way ANOVA with Tukey's correction. The Kruskal‐Wallis test was used if data did not follow a normal distribution. ns, no significance. ^∗^ *p* < 0.05, ^∗∗^ *p* < 0.01, ^∗∗∗^ *p* < 0.001. The number of independent experiments and relevant statistical methods for each panel were detailed in the figure legends.

## Conflict of Interest

The authors declare no conflict of interest.

## Author Contributions

Z.W., Z.L., and L.W. contributed equally to this work. Z.W. and Z.L. designed, performed and interpreted the experiments and wrote the manuscript. L.W., H.Z., and H.Y. contributed to building mathematical models and data analysis. X.Y., L.B., G.D., and L.R. contributed to data analysis and interpretation. Y.F. and L.L. assisted in cell experiments. S.G., Y.Z., and F.Z. assisted in animal experiments. L.Y., F.D., H.L., and Y.L. contribute to data curation and formal analysis. Sh.L., F.W., X.L. and Si.L. contribute to the experimental design, manuscript, and supervision. Sh.L. developed the original concept.

## Supporting information



Supporting Information

## Data Availability

The data that support the findings of this study are available from the corresponding author upon reasonable request.

## References

[1] C. López‐Otín , G. Kroemer , Cell 2021, 184, 33. 33340459 10.1016/j.cell.2020.11.034

[2] S. I. Inoue , M. J. Emmett , H. W. Lim , M. Midha , H. J. Richter , I. J. Celwyn , R. Mehmood , M. Chondronikola , S. Klein , A. K. Hauck , M. A. Lazar , Cell Metab. 2024, 36, 1764. 38889724 10.1016/j.cmet.2024.05.011 PMC11305953

[3] A. Zarbock , C. Schmidt , H. Van Aken , C. Wempe , S. Martens , P. K. Zahn , B. Wolf , U. Goebel , C. I. Schwer , P. Rosenberger , H. Haeberle , D. Görlich , J. A. Kellum , M. Meersch , J. Am. Med. Assoc. 2015, 313, 2133. 10.1001/jama.2015.4189 26024502

[4] N. Tapuria , Y. Kumar , M. M. Habib , M. Abu Amara , A. M. Seifalian , B. R. Davidson , J. Surg. Res. 2008, 150, 304. 19040966 10.1016/j.jss.2007.12.747

[5] D. M. Yellon , D. J. Hausenloy , N. Engl. J. Med. 2007, 357, 1121. 17855673 10.1056/NEJMra071667

[6] M. M. Nava , Y. A. Miroshnikova , L. C. Biggs , D. B. Whitefield , F. Metge , J. Boucas , H. Vihinen , E. Jokitalo , X. Li , J. M. García Arcos , B. Hoffmann , R. Merkel , C. M. Niessen , K. N. Dahl , S. A. Wickström , Cell 2020, 181, 800. 32302590 10.1016/j.cell.2020.03.052 PMC7237863

[7] F. N. Lolo , N. Walani , E. Seemann , D. Zalvidea , D. M. Pavón , G. Cojoc , M. Zamai , C. Viaris de Lesegno , F. Martínez de Benito , M. Sánchez‐Álvarez , J. J. Uriarte , A. Echarri , D. Jiménez‐Carretero , J. C. Escolano , S. A. Sánchez , V. R. Caiolfa , D. Navajas , X. Trepat , J. Guck , C. Lamaze , P. Roca‐Cusachs , M. M. Kessels , B. Qualmann , M. Arroyo , M. A. Del Pozo , Nat. Cell Biol. 2023, 25, 120. 36543981 10.1038/s41556-022-01034-3 PMC9859760

[8] J. Liu , T. Dietz , S. R. Carpenter , M. Alberti , C. Folke , E. Moran , A. N. Pell , P. Deadman , T. Kratz , J. Lubchenco , E. Ostrom , Z. Ouyang , W. Provencher , C. L. Redman , S. H. Schneider , W. W. Taylor , Science 2007, 317, 1513. 17872436 10.1126/science.1144004

[9] R. R. Pompano , A. H. Chiang , C. J. Kastrup , R. F. Ismagilov , Annu. Rev. Biochem. 2017, 86, 333. 28654324 10.1146/annurev-biochem-060815-014207 PMC10852032

[10] A. L. Hodgkin , A. F. Huxley , J. Physiol. 1952, 116, 449. 14946713 10.1113/jphysiol.1952.sp004717 PMC1392213

[11] K. Kikuchi , L. Galera‐Laporta , C. Weatherwax , J. Y. Lam , E. C. Moon , E. A. Theodorakis , J. Garcia‐Ojalvo , G. M. Süel , Science 2022, 378, 43. 36201591 10.1126/science.abl7484 PMC10593254

[12] B. Z. Jia , Y. Qi , J. D. Wong‐Campos , S. G. Megason , A. E. Cohen , Nature 2023, 622, 149. 37758945 10.1038/s41586-023-06561-z

[13] C. C. Chen , L. Wang , M. V. Plikus , T. X. Jiang , P. J. Murray , R. Ramos , C. F. Guerrero‐Juarez , M. W. Hughes , O. K. Lee , S. Shi , R. B. Widelitz , A. D. Lander , C. M. Chuong , Cell 2015, 161, 277. 25860610 10.1016/j.cell.2015.02.016 PMC4393531

[14] H. S. Wong , K. Park , A. Gola , A. P. Baptista , C. H. Miller , D. Deep , M. Lou , L. F. Boyd , A. Y. Rudensky , P. A. Savage , G. Altan‐Bonnet , J. S. Tsang , R. N. Germain , Cell 2021, 184, 3981. 34157301 10.1016/j.cell.2021.05.028 PMC8390950

[15] J. P. Higgins , Yale J. Biol. Med. 2002, 75, 247. 14580107 PMC2588816

[16] C. P. Heisenberg , Y. Bellaïche , Cell 2013, 153, 948. 23706734 10.1016/j.cell.2013.05.008

[17] X. Di , X. Gao , L. Peng , J. Ai , X. Jin , S. Qi , H. Li , K. Wang , D. Luo , Signal Transduction Targeted Ther. 2023, 8, 282. 10.1038/s41392-023-01501-9 PMC10387486 37518181

[18] N. Marinval , S. Y. Chew , APL Bioeng. 2021, 5, 021505. 33948526 10.1063/5.0037814 PMC8088332

[19] Y. Long , Y. Niu , K. Liang , Y. Du , Trends Cell Biol. 2022, 32, 70. 34810063 10.1016/j.tcb.2021.10.002

[20] W. Wei , F. Rao , F. Liu , Y. Xue , C. Deng , Z. Wang , J. Zhu , H. Yang , X. Li , M. Zhang , Y. Fu , W. Zhu , Z. Shan , S. Wu , J. Cell. Physiol. 2018, 233, 4981. 29215718 10.1002/jcp.26337

[21] C. Liu , X. Gao , Y. Li , W. Sun , Y. Xu , Y. Tan , R. Du , G. Zhong , D. Zhao , Z. Liu , X. Jin , Y. Zhao , Y. Wang , X. Yuan , J. Pan , G. Yuan , Y. Li , W. Xing , G. Kan , Y. Wang , Q. Li , X. Han , J. Li , S. Ling , Y. Li , Bone Res. 2022, 10, 18. 35210394 10.1038/s41413-022-00191-3 PMC8873336

[22] K. Shiraishi , P. P. Shah , M. P. Morley , C. Loebel , G. T. Santini , J. Katzen , M. C. Basil , S. M. Lin , J. D. Planer , E. Cantu , D. L. Jones , A. N. Nottingham , S. Li , F. L. Cardenas‐Diaz , S. Zhou , J. A. Burdick , R. Jain , E. E. Morrisey , Cell 2023, 186, 1478. 36870331 10.1016/j.cell.2023.02.010 PMC10065960

[23] N. Migulina , B. Kelley , E. Y. Zhang , C. M. Pabelick , Y. S. Prakash , E. R. Vogel , Compr. Physiol. 2023, 13, 5157. 37770188 10.1002/cphy.c230006

[24] L. Gattinoni , T. Tonetti , M. Cressoni , P. Cadringher , P. Herrmann , O. Moerer , A. Protti , M. Gotti , C. Chiurazzi , E. Carlesso , D. Chiumello , M. Quintel , Intens. Care Med. 2016, 42, 1567. 10.1007/s00134-016-4505-2 27620287

[25] L. Pinhu , T. Whitehead , T. Evans , M. Griffiths , Lancet 2003, 361, 332. 12559881 10.1016/S0140-6736(03)12329-X

[26] E. Fan , D. Brodie , A. S. Slutsky , J. Am. Med. Assoc. 2018, 319, 698. 10.1001/jama.2017.21907 29466596

[27] D. B. Mahat , H. H. Salamanca , F. M. Duarte , C. G. Danko , J. T. Lis , Mol. Cell 2016, 62, 63. 27052732 10.1016/j.molcel.2016.02.025 PMC4826300

[28] U. Kersting , L. Wojnowski , W. Steigner , H. Oberleithner , Kidney Int. 1991, 39, 891. 2067205 10.1038/ki.1991.112

[29] O. Chaudhuri , L. Gu , M. Darnell , D. Klumpers , S. A. Bencherif , J. C. Weaver , N. Huebsch , D. J. Mooney , Nat. Commun. 2015, 6, 6364. 10.1038/ncomms7365 PMC4518451 25695512

[30] M. Guo , A. F. Pegoraro , A. Mao , E. H. Zhou , P. R. Arany , Y. Han , D. T. Burnette , M. H. Jensen , K. E. Kasza , J. R. Moore , F. C. Mackintosh , J. J. Fredberg , D. J. Mooney , J. Lippincott‐Schwartz , D. A. Weitz , Proc. Natl. Acad. Sci. USA 2017, 114, E8618. 28973866 10.1073/pnas.1705179114 PMC5642688

[31] J. Liu , C. Zhao , X. Xiao , A. Li , Y. Liu , J. Zhao , L. Fan , Z. Liang , W. Pang , W. Yao , W. Li , J. Zhou , Nat. Commun. 2023, 14, 6457. 37833282 10.1038/s41467-023-42341-z PMC10576099

[32] W. Li , X. Yu , F. Xie , B. Zhang , S. Shao , C. Geng , A. U. R. Aziz , X. Liao , B. Liu , iScience 2018, 7, 180. 30267679 10.1016/j.isci.2018.09.002 PMC6153118

[33] Y. Zhang , Y. Qi , J. J. Li , W. J. He , X. H. Gao , Y. Zhang , X. Sun , J. Tong , J. Zhang , X. L. Deng , X. J. Du , W. Xie , Cardiovasc. Res. 2021, 117, 1091. 32531044 10.1093/cvr/cvaa163

[34] H. Kitano , Nat. Rev. Genet. 2004, 5, 826. 15520792 10.1038/nrg1471

[35] M. Scheffer , S. R. Carpenter , T. M. Lenton , J. Bascompte , W. Brock , V. Dakos , J. van de Koppel , I. A. van de Leemput , S. A. Levin , E. H. van Nes , M. Pascual , J. Vandermeer , Science 2012, 338, 344. 23087241 10.1126/science.1225244

[36] A. Becskei , B. Séraphin , L. Serrano , EMBO J. 2001, 20, 2528. 11350942 10.1093/emboj/20.10.2528 PMC125456

[37] M. I. Stefan , N. Le Novère , PLoS Comput. Biol. 2013, 9, 1003106. 10.1371/journal.pcbi.1003106 PMC3699289 23843752

[38] A. V. Hill , J. Physiol. 1910, 40, 190. 16993004 10.1113/jphysiol.1910.sp001366 PMC1533746

[39] M. Endo , Physiol. Rev. 2009, 89, 1153. 19789379 10.1152/physrev.00040.2008

[40] M. Wullschleger , J. Blanch , M. Egger , Cardiovasc. Res. 2017, 113, 542. 28158491 10.1093/cvr/cvx020

[41] M. Carlström , C. S. Wilcox , W. J. Arendshorst , Physiol. Rev. 2015, 95, 405. 25834230 10.1152/physrev.00042.2012 PMC4551215

[42] A. J. Morgan , R. Jacob , Biochem. J. 1994, 300, 665. 8010948 10.1042/bj3000665 PMC1138219

[43] T. J. Jentsch , Nat. Rev. Mol. Cell Biol. 2016, 17, 293. 27033257 10.1038/nrm.2016.29

[44] E. K. Hoffmann , I. H. Lambert , S. F. Pedersen , Physiol. Rev. 2009, 89, 193. 19126758 10.1152/physrev.00037.2007

[45] F. Lang , G. L. Busch , M. Ritter , H. Völkl , S. Waldegger , E. Gulbins , D. Häussinger , Physiol. Rev. 1998, 78, 247. 9457175 10.1152/physrev.1998.78.1.247

[46] A. Schwab , A. Fabian , P. J. Hanley , C. Stock , Physiol. Rev. 2012, 92, 1865. 23073633 10.1152/physrev.00018.2011

[47] Y. Wang , Z. Sun , J. Ping , J. Tang , B. He , T. Chang , Q. Zhou , S. Yuan , Z. Tang , X. Li , Y. Lu , R. He , X. He , Z. Liu , L. Yin , N. Wu , Nat. Commun. 2023, 14, 7075. 37925509 10.1038/s41467-023-42817-y PMC10625614

[48] X. Yang , C. Lin , X. Chen , S. Li , X. Li , B. Xiao , Nature 2022, 604, 377. 35388220 10.1038/s41586-022-04574-8

[49] K. M. Beverley , I. Levitan , Front. Cell Dev. Biol. 2024, 12, 1352259. 38333595 10.3389/fcell.2024.1352259 PMC10850386

[50] I. Levitan , A. E. Christian , T. N. Tulenko , G. H. Rothblat , J. Gen. Physiol. 2000, 115, 405. 10736308 10.1085/jgp.115.4.405 PMC2233759

[51] T. K. Klausen , C. Hougaard , E. K. Hoffmann , S. F. Pedersen , Am. J. Physiol. Cell Physiol. 2006, 291, C757. 16687471 10.1152/ajpcell.00029.2006

[52] K. Lei , A. Kurum , M. Kaynak , L. Bonati , Y. Han , V. Cencen , M. Gao , Y. Q. Xie , Y. Guo , M. T. M. Hannebelle , Y. Wu , G. Zhou , M. Guo , G. E. Fantner , M. S. Sakar , L. Tang , Nat. Biomed. Eng. 2021, 5, 1411. 34873307 10.1038/s41551-021-00826-6 PMC7612108

[53] D. Y. Litvinov , E. V. Savushkin , A. D. Dergunov , J. Lipids 2018, 2018, 3965054. 30174957 10.1155/2018/3965054 PMC6106919

[54] Z. Yang , Y. Huo , S. Zhou , J. Guo , X. Ma , T. Li , C. Fan , L. Wang , Cell Metab. 2022, 34, 2018. 36351432 10.1016/j.cmet.2022.10.010

[55] L. Debbi , S. Guo , D. Safina , S. Levenberg , Biotechnol. Adv. 2022, 59, 107983. 35588952 10.1016/j.biotechadv.2022.107983 PMC9420194

[56] H. Kuang , G. Dou , L. Cheng , X. Wang , H. Xu , X. Liu , F. Ding , X. Yang , S. Liu , L. Bao , H. Liu , Y. Liu , B. Li , Y. Jin , S. Liu , Nat. Metab. 2023, 5, 111. 36658400 10.1038/s42255-022-00723-5

[57] E. R. Abels , X. O. Breakefield , Cell. Mol. Neurobiol. 2016, 36, 301. 27053351 10.1007/s10571-016-0366-z PMC5546313

[58] S. Liu , D. Liu , C. Chen , K. Hamamura , A. Moshaverinia , R. Yang , Y. Liu , Y. Jin , S. Shi , Cell Metab. 2015, 22, 606. 26365178 10.1016/j.cmet.2015.08.018 PMC4731233

[59] J. M. Whitlock , H. C. Hartzell , Annu. Rev. Physiol. 2017, 79, 119. 27860832 10.1146/annurev-physiol-022516-034031 PMC5556385

[60] A. Kira , I. Tatsutomi , K. Saito , M. Murata , I. Hattori , H. Kajita , N. Muraki , Y. Oda , S. Satoh , Y. Tsukamoto , S. Kimura , K. Onoue , S. Yonemura , S. Arakawa , H. Kato , T. Hirashima , K. Kawane , Dev. Cell 2023, 58, 1282. 37315563 10.1016/j.devcel.2023.05.008

[61] L. G. Lima , R. Chammas , R. Q. Monteiro , M. E. Moreira , M. A. Barcinski , Cancer Lett. 2009, 283, 168. 19401262 10.1016/j.canlet.2009.03.041

[62] J. Suzuki , M. Umeda , P. J. Sims , S. Nagata , Nature 2010, 468, 834. 21107324 10.1038/nature09583

[63] T. Fujii , A. Sakata , S. Nishimura , K. Eto , S. Nagata , Proc. Natl. Acad. Sci. USA 2015, 112, 12800. 26417084 10.1073/pnas.1516594112 PMC4611630

[64] N. Wu , V. Cernysiov , D. Davidson , H. Song , J. Tang , S. Luo , Y. Lu , J. Qian , I. E. Gyurova , S. N. Waggoner , V. Q. Trinh , R. Cayrol , A. Sugiura , H. M. McBride , J. F. Daudelin , N. Labrecque , A. Veillette , Cell Rep. 2020, 30, 1129. 31995754 10.1016/j.celrep.2019.12.066 PMC7104872

[65] Y. Hu , J. H. Kim , K. He , Q. Wan , J. Kim , M. Flach , T. Kirchhausen , A. Vortkamp , F. Winau , J. Exp. Med. 2016, 213, 2759. 27810927 10.1084/jem.20160612 PMC5110022

[66] C. A. Juul , S. Grubb , K. A. Poulsen , T. Kyed , N. Hashem , I. H. Lambert , E. H. Larsen , E. K. Hoffmann , Pflugers Arch. 2014, 466, 1899. 24419539 10.1007/s00424-013-1428-4 PMC4159566

[67] R. Centeio , J. Ousingsawat , R. Schreiber , K. Kunzelmann , Front. Cell Dev. Biol. 2020, 8, 596879. 33335902 10.3389/fcell.2020.596879 PMC7736618

[68] R. Benedetto , L. Sirianant , I. Pankonien , P. Wanitchakool , J. Ousingsawat , I. Cabrita , R. Schreiber , M. Amaral , K. Kunzelmann , Pflugers Arch. 2016, 468, 1751. 27514381 10.1007/s00424-016-1862-1

[69] J. Gao , B. Barzel , A. L. Barabási , Nature 2016, 530, 307. 26887493 10.1038/nature16948

[70] G. Forzieri , V. Dakos , N. G. McDowell , A. Ramdane , A. Cescatti , Nature 2022, 608, 534. 35831499 10.1038/s41586-022-04959-9 PMC9385496

[71] Y. Zhang , T. Vu , D. C. Palmer , R. J. Kishton , L. Gong , J. Huang , T. Nguyen , Z. Chen , C. Smith , F. Livák , R. Paul , C. P. Day , C. Wu , G. Merlino , K. Aldape , X. Y. Guan , P. Jiang , Nat. Med. 2022, 28, 1421. 35501486 10.1038/s41591-022-01799-y PMC9406236

[72] A. Echarri , D. M. Pavón , S. Sánchez , M. García‐García , E. Calvo , C. Huerta‐López , D. Velázquez‐Carreras , C. Viaris de Lesegno , N. Ariotti , A. Lázaro‐Carrillo , R. Strippoli , D. De Sancho , J. Alegre‐Cebollada , C. Lamaze , R. G. Parton , M. A. Del Pozo , Nat. Commun. 2019, 10, 5828. 31862885 10.1038/s41467-019-13782-2 PMC6925243

[73] L. Dai , D. Vorselen , K. S. Korolev , J. Gore , Science 2012, 336, 1175. 22654061 10.1126/science.1219805

[74] A. D. Barnosky , E. A. Hadly , J. Bascompte , E. L. Berlow , J. H. Brown , M. Fortelius , W. M. Getz , J. Harte , A. Hastings , P. A. Marquet , N. D. Martinez , A. Mooers , P. Roopnarine , G. Vermeij , J. W. Williams , R. Gillespie , J. Kitzes , C. Marshall , N. Matzke , D. P. Mindell , E. Revilla , A. B. Smith , Nature 2012, 486, 52. 22678279 10.1038/nature11018

[75] X. Shen , C. Wang , X. Zhou , W. Zhou , D. Hornburg , S. Wu , M. P. Snyder , Nat. Aging 2024, 4, 1619. 39143318 10.1038/s43587-024-00692-2 PMC11564093

[76] S. Bhattacharya , R. B. Conolly , N. E. Kaminski , R. S. Thomas , M. E. Andersen , Q. Zhang , Toxicol. Sci. 2010, 115, 51. 20123757 10.1093/toxsci/kfq035 PMC2855357

[77] L. Wang , B. L. Walker , S. Iannaccone , D. Bhatt , P. J. Kennedy , W. T. Tse , Proc. Natl. Acad. Sci. USA 2009, 106, 6638. 19366677 10.1073/pnas.0806137106 PMC2672527

[78] M. G. Olde Rikkert , V. Dakos , T. G. Buchman , R. Boer , L. Glass , A. O. Cramer , S. Levin , E. van Nes , G. Sugihara , M. D. Ferrari , E. A. Tolner , I. van de Leemput , J. Lagro , R. Melis , M. Scheffer , Crit. Care Med. 2016, 44, 601. 26765499 10.1097/CCM.0000000000001564

[79] G. Chiosis , C. S. Digwal , J. B. Trepel , L. Neckers , Nat. Rev. Mol. Cell Biol. 2023, 24, 797. 37524848 10.1038/s41580-023-00640-9 PMC10592246

[80] J. L. Watson , E. Seinkmane , C. T. Styles , A. Mihut , L. K. Krüger , K. E. McNally , V. J. Planelles‐Herrero , M. Dudek , P. M. McCall , S. Barbiero , M. Vanden Oever , S. Y. Peak‐Chew , B. T. Porebski , A. Zeng , N. M. Rzechorzek , D. C. S. Wong , A. D. Beale , A. Stangherlin , M. Riggi , J. Iwasa , J. Morf , C. Miliotis , A. Guna , A. J. Inglis , J. Brugués , R. M. Voorhees , J. E. Chambers , Q. J. Meng , J. S. O'Neill , R. S. Edgar , et al., Nature 2023, 623, 842. 37853127 10.1038/s41586-023-06626-z PMC10665201

[81] D. J. Hausenloy , D. M. Yellon , Nat. Rev. Cardiol. 2016, 13, 193. 26843289 10.1038/nrcardio.2016.5

[82] Xu , A. D. Chisholm , Curr. Biol. 2011, 21, 1960. 22100061 10.1016/j.cub.2011.10.050 PMC3237753

[83] M. Høyer‐Hansen , M. Jäättelä , Cell Death Differ. 2007, 14, 1576. 17612585 10.1038/sj.cdd.4402200

[84] P. Wales , C. E. Schuberth , R. Aufschnaiter , J. Fels , I. García‐Aguilar , A. Janning , C. P. Dlugos , M. Schäfer‐Herte , C. Klingner , M. Wälte , J. Kuhlmann , E. Menis , H. Kang , R. Wedlich‐Söldner , Elife 2016, 5, 19850. 10.7554/eLife.19850 PMC5140269 27919320

[85] J. Jesty , E. Beltrami , Arterioscler Thromb. Vasc. Biol. 2005, 25, 2463. 16179597 10.1161/01.ATV.0000187463.91403.b2

[86] M. McLean , R. Smith , Reproduction 2001, 121, 493. 11277868 10.1530/rep.0.1210493

[87] J. H. Jaggar , V. A. Porter , W. J. Lederer , M. T. Nelson , Am. J. Physiol. Cell Physiol. 2000, 278, C235. 10666018 10.1152/ajpcell.2000.278.2.C235

[88] Y. Li , Q. Zhang , Z. Liu , C. Wang , S. Han , Q. Ma , W. Du , Brief Bioinf. 2021, 22, bbaa354. 10.1093/bib/bbaa354 PMC8294561 33367506

[89] F. Melo‐Fonseca , O. Carvalho , M. Gasik , G. Miranda , F. S. Silva , Bio‐Des. Manuf. 2023, 6, 340.

[90] C. M. Bobba , Q. Fei , V. Shukla , H. Lee , P. Patel , R. K. Putman , C. Spitzer , M. Tsai , M. D. Wewers , R. J. Lee , J. W. Christman , M. N. Ballinger , S. N. Ghadiali , J. A. Englert , Nat. Commun. 2021, 12, 289. 33436554 10.1038/s41467-020-20449-w PMC7804938

[91] R. Crescitelli , C. Lässer , J. Lötvall , Nat. Protoc. 2021, 16, 1548. 33495626 10.1038/s41596-020-00466-1

[92] Y. Okabe , N. Murakoshi , N. Kurebayashi , H. Inoue , Y. Ito , T. Murayama , C. Miyoshi , H. Funato , K. Ishii , D. Xu , K. Tajiri , R. Qin , K. Aonuma , Y. Murakata , Z. Song , S. Wakana , U. Yokoyama , T. Sakurai , K. Aonuma , M. Ieda , M. Yanagisawa , Proc. Natl. Acad. Sci. USA 2024, 121, 2218204121. 10.1073/pnas.2218204121 PMC11047072 38621141

[93] S. Yang , M. Golkaram , S. Oh , Y. Oh , Y. Cho , J. Yoe , S. Ju , M. A. Lalli , S. Y. Park , Y. Lee , J. Jang , Nat. Cell Biol. 2024, 26, 903. 38702503 10.1038/s41556-024-01415-w PMC11178500

