# Supplementary material for: Cellular Adaptation to Mechanical Stress Emerges via Cell Shrinkage Triggered by Nonlinear Calcium Elevation
Source: Adv Sci (Weinh). 2025 Jul 18;12(38):e03659. doi: 10.1002/advs.202503659 (PMC12520526; doi:10.1002/advs.202503659)
Supplement: Supplementary file 1 — Supporting Information [file ADVS-12-e03659-s001.docx]

**Supplementary Figures**

**
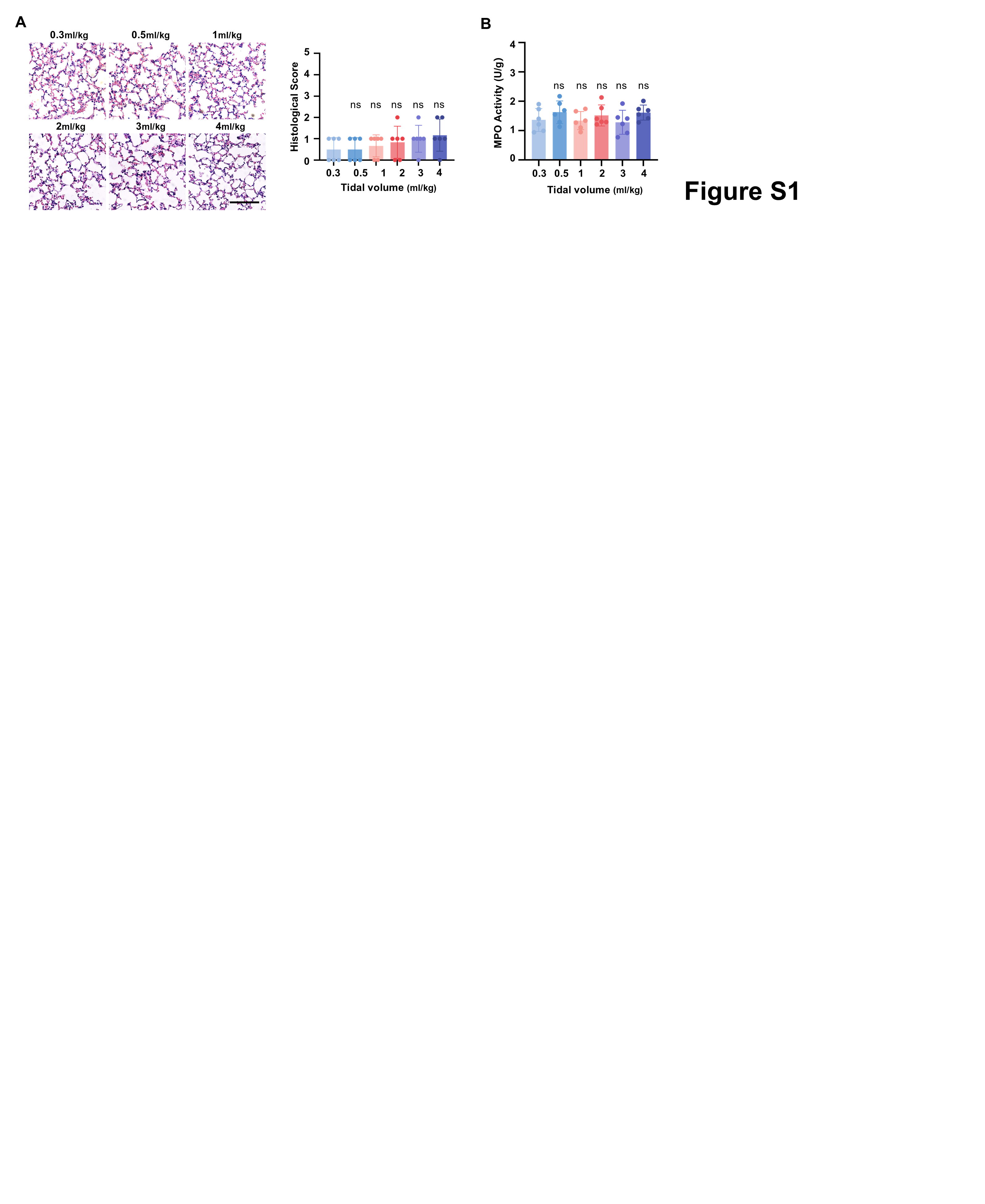
**

**Figure S1. Mild low tidal volume ventilation barely causes lung injury**

**(A-B)** Mice from the untreated control group, and mild ventilation at 0.3-4 ml/kg groups were used for subsequent analysis. **(A)** H&E staining of representative lung sections and the histological scores. Scale bar, 50 µm. n = 6 mice. **(B)** MPO activity of lung tissues. n = 6 mice. Data are presented as mean ± s.d. Statistical significance was assessed by one-way analysis of variance (ANOVA) with Tukey’s post hoc test **(A-B).**


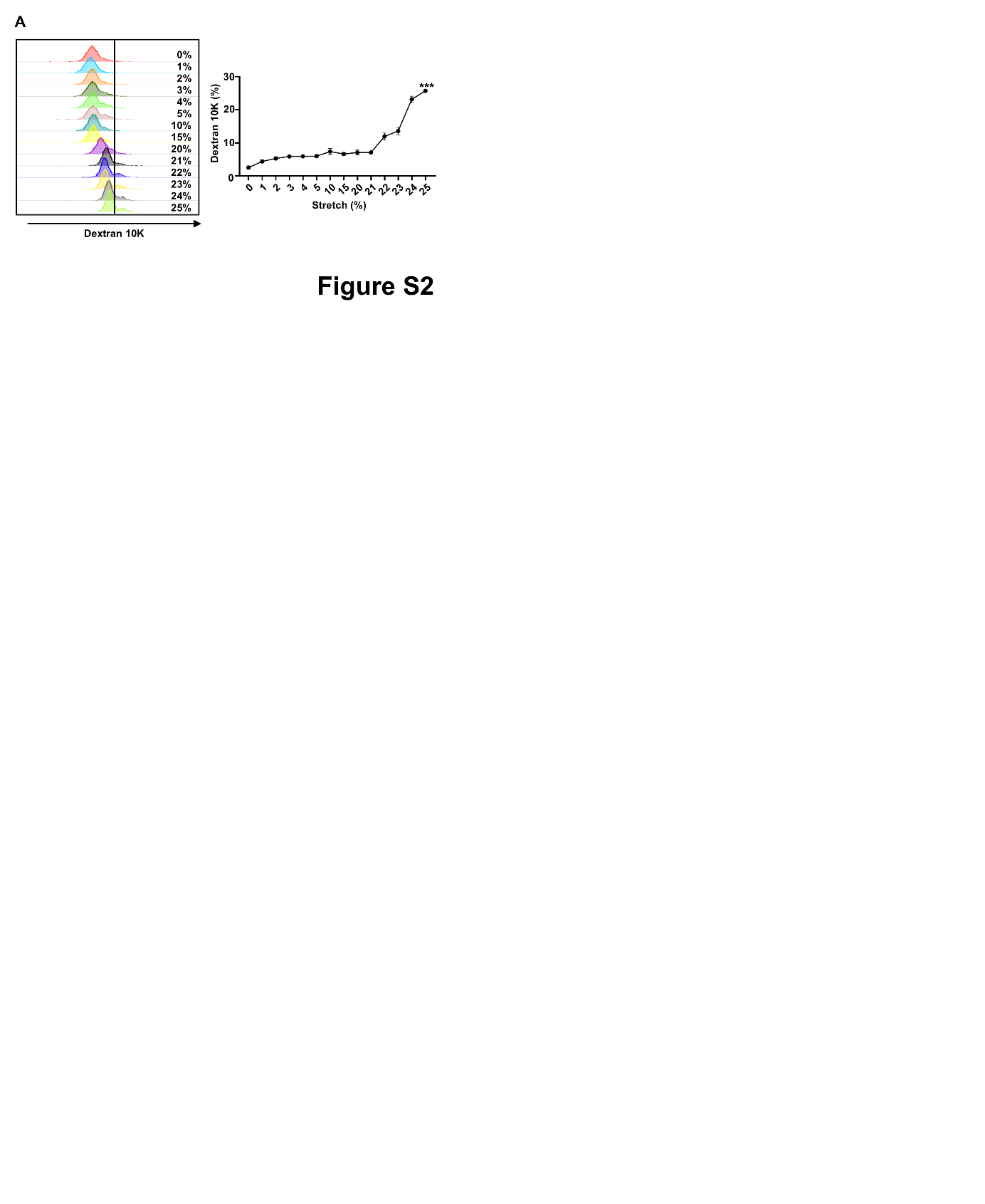


**Figure S2. Characteristics of mechanical adaptation emergence**

**(A)** Representative histograms and quantitative analysis of Dextran 10K-FITC penetration in BEAS-2b cells subjected to varying stretch amplitudes. n = 3 biologically independent samples. Data are presented as the mean ± s.d. Statistical significance was assessed by one-way ANOVA with Tukey’s post hoc test.


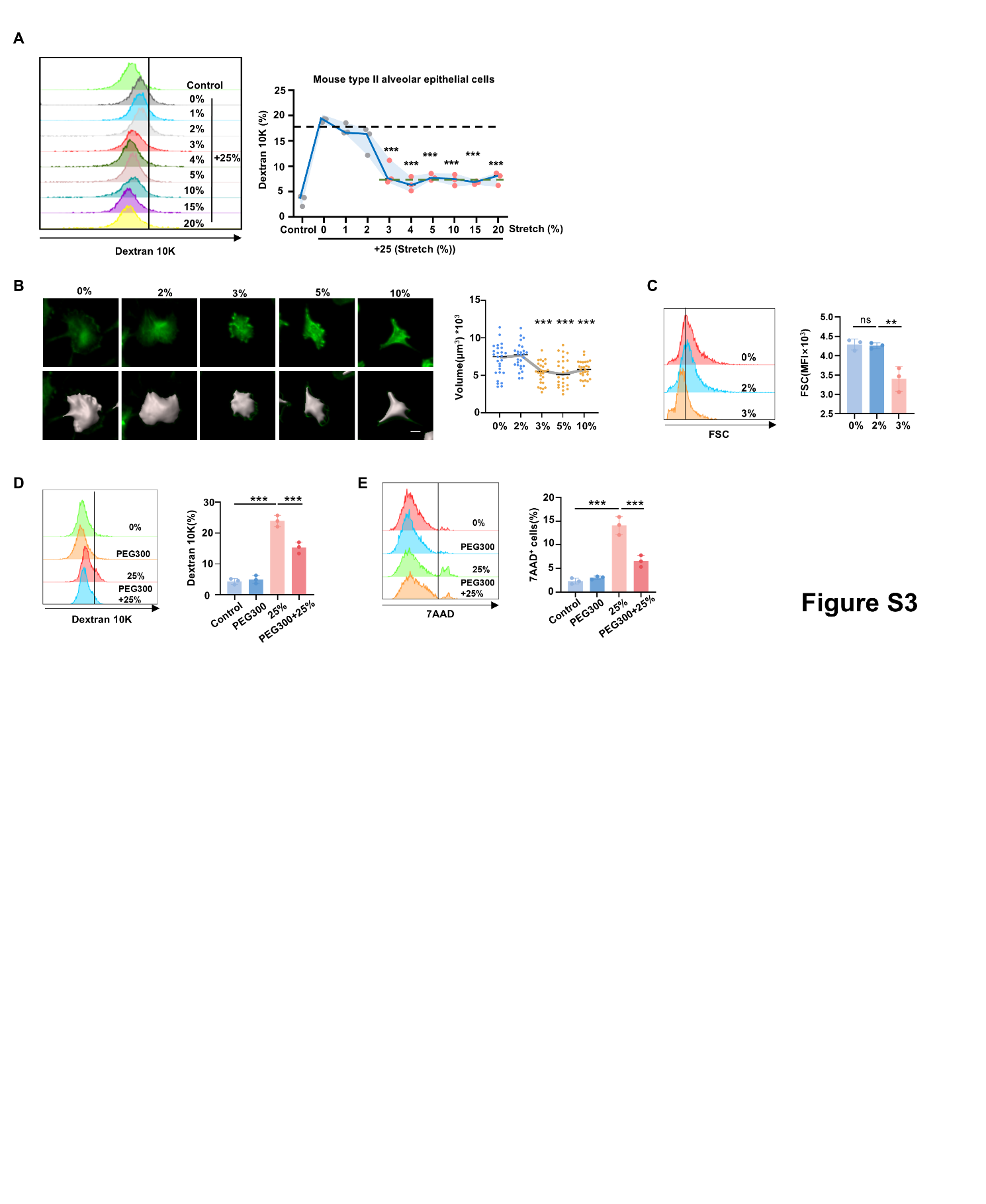
**Figure S3. Mouse alveolar epithelial cells adapt to damaging high-amplitude stretch through cell shrinkage in a nonlinear pattern**

**(A)** Representative histogram and quantitative analysis of penetration of Dextran10k-FITC in mouse type II alveolar epithelial cells following mild stretches of different amplitudes and subsequent stretch at the amplitude of 25%. n = 3 biologically independent samples. **(B)** Representative fluorescence images and corresponding 3D images of ZsGreen-transfected cells captured by CLSM and 3D reconstructed by Imaris software, with quantification of cell volume calculated by Imaris. Scale bar, 10 µm. n ≥ 25 cells per group from three independent experiments. **(C)** Cell volume detected by forward scatter (FSC) on ﬂow cytometry, n = 3 biologically independent samples. (D-E) Representative histograms of Dextran 10K-FITC penetration **(D)** and 7AAD staining **(E)** in mouse alveolar epithelial cells treated with 1% PEG300 prior to 25% stretch and quantitative analysis. n = 3 biologically independent samples. Data are presented as the mean ± s.d. Statistical significance was assessed by one-way ANOVA with Tukey’s post hoc test (A, C-E) and Kruskal-Wallis test (B).

**
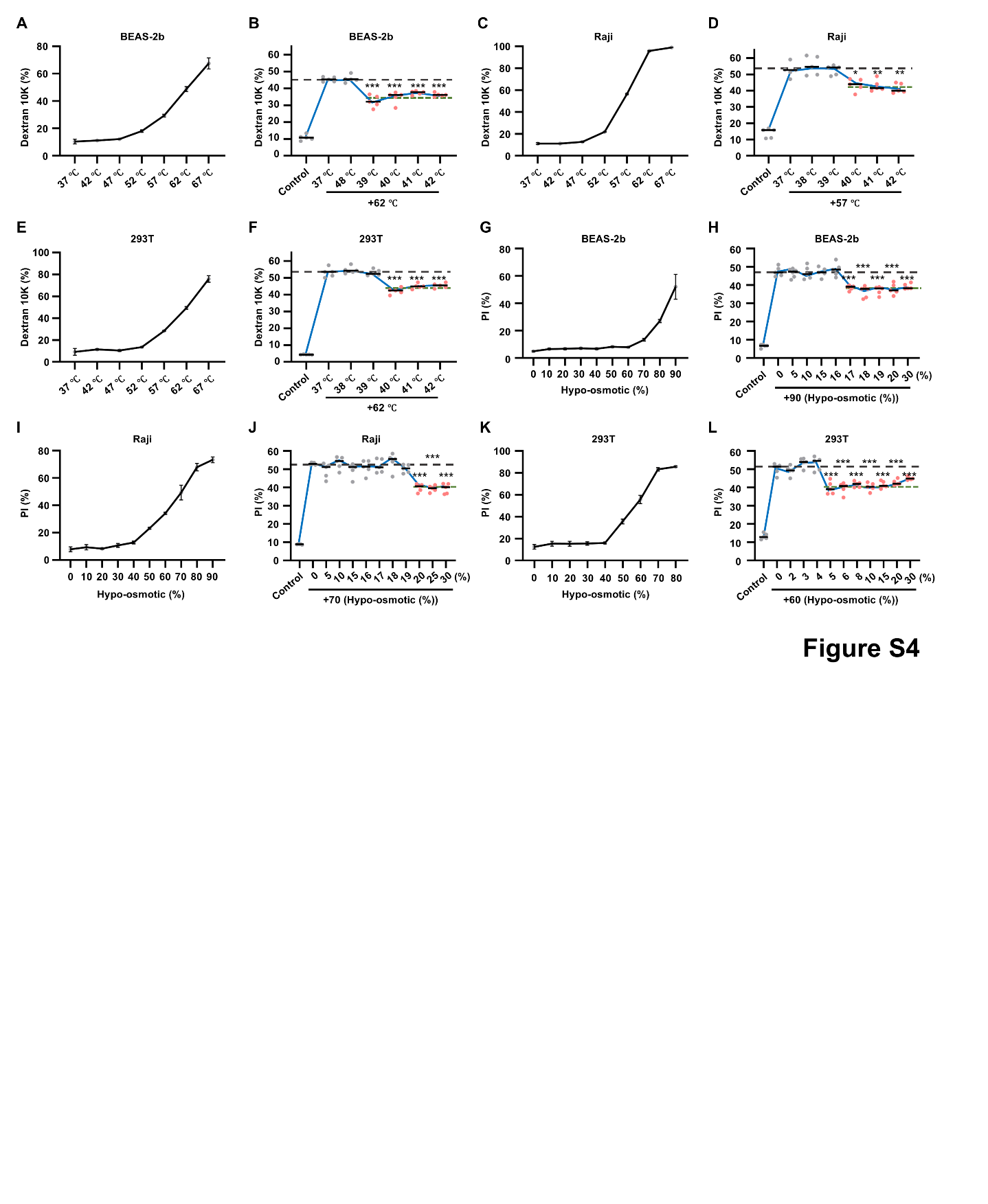
**

**Figure S4. Cellular adaptation against hypo-osmotic and heat stress emerges in nonlinear manner**

**(A, C, E)** Quantitative analysis of Dextran 10K-FITC penetration in BEAS-2b cells **(A)**, Raji cells **(C)**, or 293T cells **(E)** subjected to heat stress at varying temperatures, as detected by flow cytometry to evaluate cell damage. n = 3 biologically independent samples. **(B, D, F)** Quantitative analysis of Dextran 10K-FITC penetration in BEAS-2b cells **(B)**, Raji cells **(D)**, or 293T cells **(F)** treated with different mild heat stresses followed by damaging heat stress at a temperature that caused damage to approximately 50% of the cells. n = 5 biologically independent samples. **(G, I, K)** Quantitative analysis of PI positive BEAS-2b cells **(G)**, Raji cells **(I)**, or 293T cells **(K)** after treatment with hypo-osmotic stress at varying level, as detected by flow cytometry to evaluate cell damage. n = 3 biologically independent samples. **(H, J, L)** Quantitative analysis of PI positive BEAS-2b cells **(H)**, Raji cells **(J)**, or 293T cells **(L)** after pretreatment with varying mild hypo-osmotic stress and following damaging hypo-osmotic stress that induced approximately 50% of cell damage. n = 5 biologically independent samples. Data are presented as the mean ± s.d. Statistical significance was assessed by one-way ANOVA with Tukey’s post hoc test **(A-L)**.


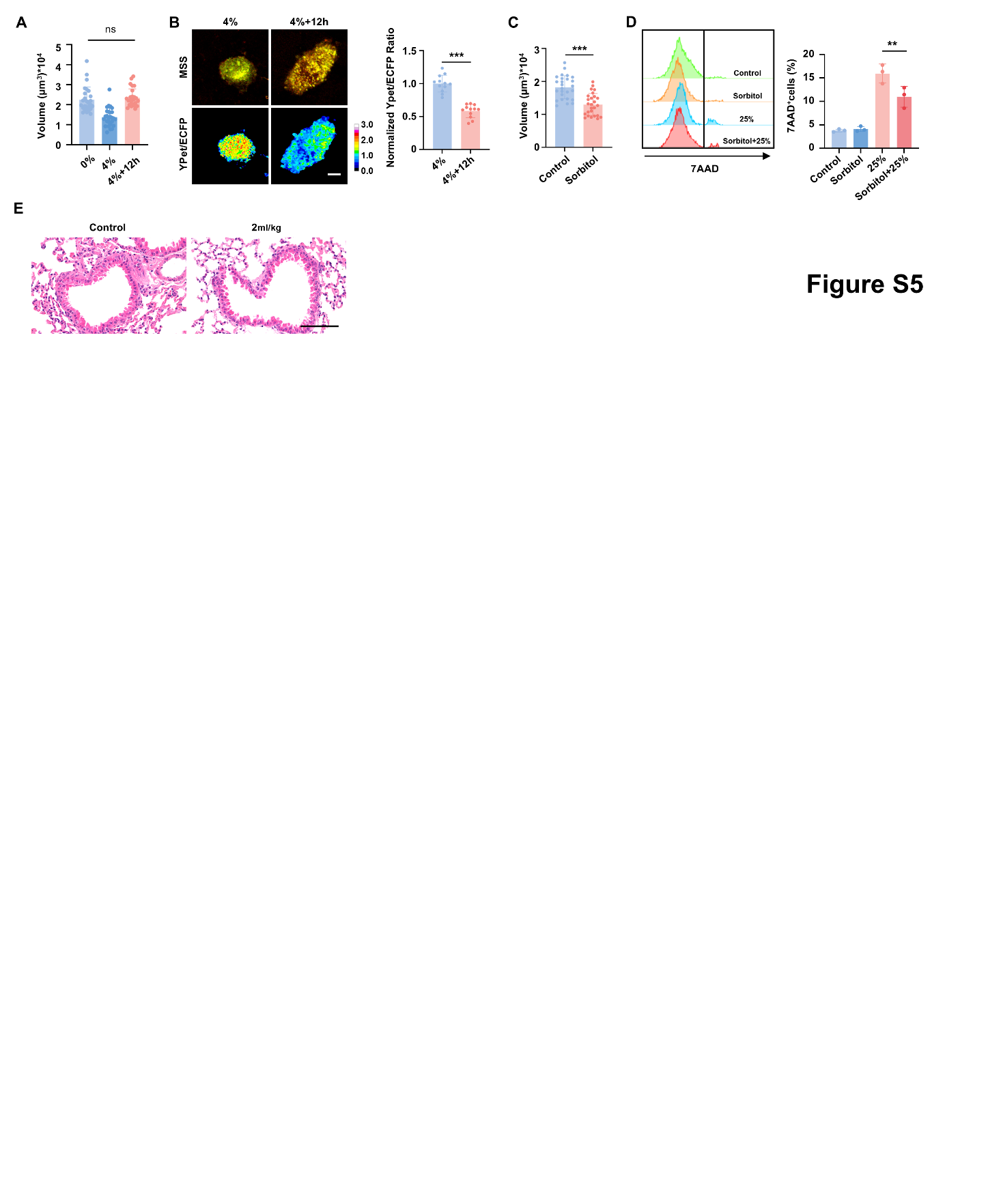


**Figure S5. Cell shrinkage occurred in mechanical adaptive state**

**(A)** Cell volume analysis immediately after 4% stretch and 12 hours after 4% stretch. n > 25 cells from three independent experiments. **(B)** Representative ﬂuorescence images of MSS-expressing cell showing YPet/ECFP emission ratio immediately after 4% stretch and 12 hours after stretch. Scale bar, 10 µm. n = 12 biologically independent samples. **(C)** Volume of BEAS-2b cells under osmotic compression with sorbitol. n > 25 cells from three independent experiments. **(D)** Representative histograms of 7AAD staining in BEAS-2b cells treated with sorbitol prior to 25% stretch and quantitative analysis. n = 3 biologically independent samples. **(E)** H&E staining of representative lung sections from untreated mice and mice treated with ventilation at 2 ml/kg. Scale bar, 75 µm. n = 6 mice. Data are presented as the mean ± s.d. Statistical significance was assessed by one-way ANOVA with Tukey’s post hoc test **(A, D)** and unpaired two-tailed Student’s t-test **(B, C)**.

**
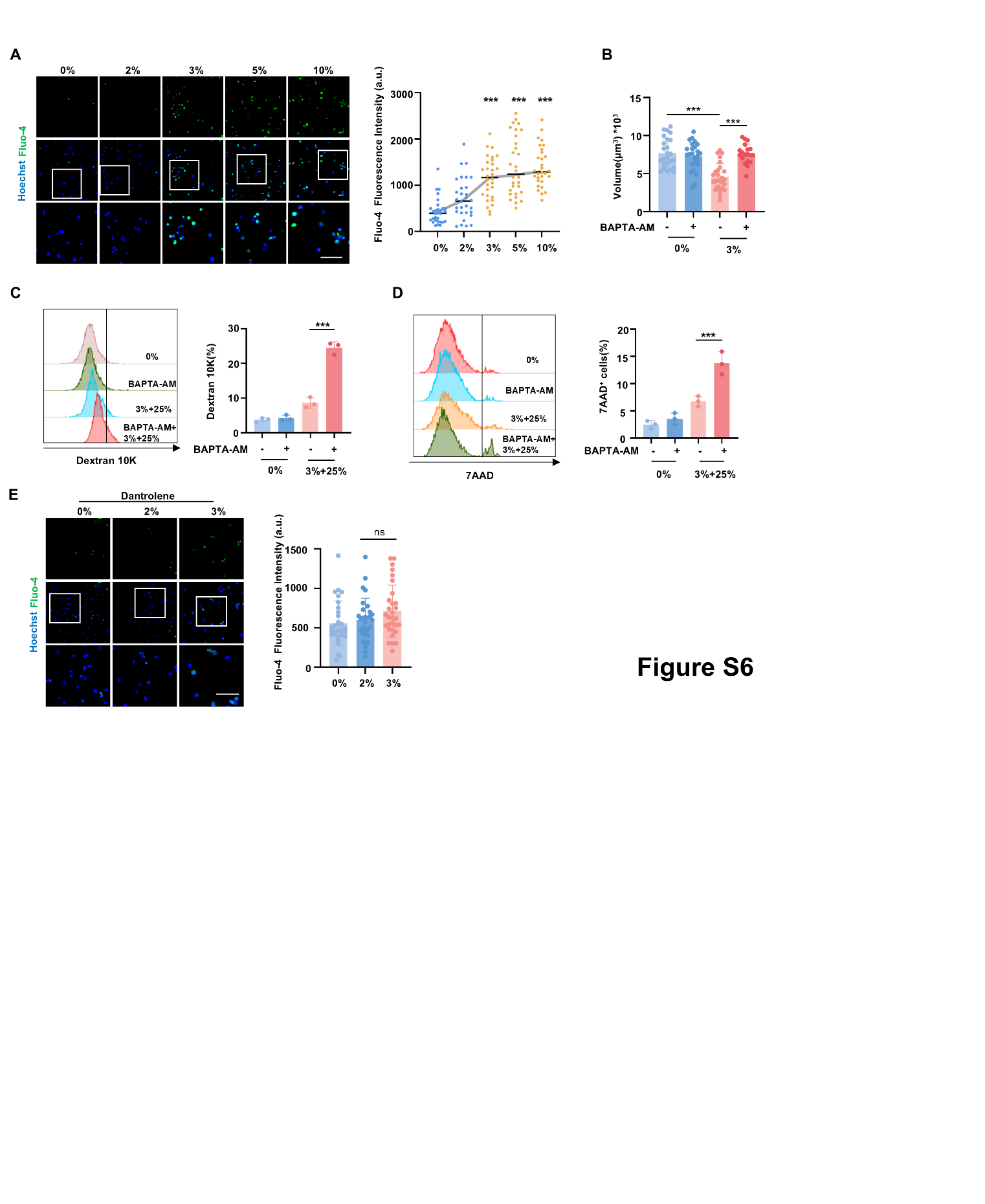
**

**Figure S6. RYR-mediated ER calcium release triggers nonlinear cell volume decrease in alveolar epithelial cells**

**(A)** Representative ﬂuorescence images of Ca^2+^ sensor dye Fluo-4-AM and quantitative fluorescence intensity in cells detected by CLSM after stretches of varying amplitudes. n ≥ 30 cells per group from three independent experiments. Scale bar, 50μm. **(B)** The volume of alveolar epithelial cells pretreated with Ca^2+^ chelator BAPTA-AM prior to 3% stretch, analyzed by Imaris. n ≥ 25 cells per group from three independent experiments. **(C-D)** Representative histograms of Dextran 10K-FITC penetration **(C)** and 7AAD staining **(D)** in alveolar epithelial cells pretreated with BAPTA-AM following the process of 3%+25% stretch, with quantitative analysis. n = 3 biologically independent samples. **(E)** Representative ﬂuorescence images of Ca^2+^ and quantitative fluorescence intensity of stretched cells pretreated with RYR inhibitor Dantrolene. n ≥ 30 cells per group from three independent experiments. Scale bar, 50µm. Data are presented as the mean ± s.d. Statistical significance was assessed by one-way ANOVA with Tukey’s post hoc test (B-D) and Kruskal-Wallis test (A, E).


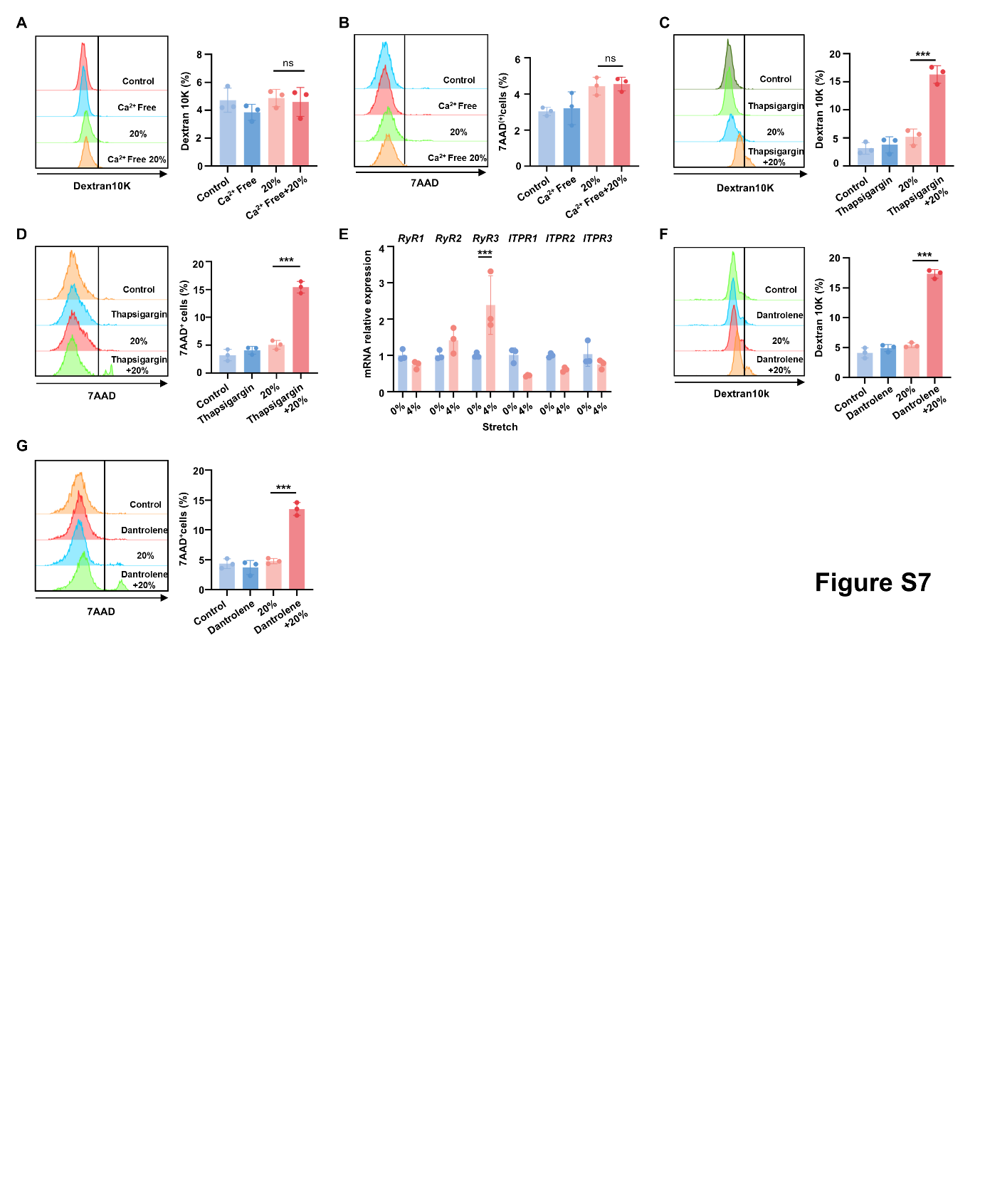


**Figure S7. The effects of different sources-derived Ca^2+^ on mechanical adaptation emergence**

**(A-B)** Representative histograms of Dextran 10K-FITC penetration **(A)** or 7AAD staining **(B)** in 20% stretched BEAS-2b cells cultured in Ca^2+^ free medium. n = 3 biologically independent samples. **(C-D)** Representative histograms of Dextran 10K-FITC penetration **(C)** or 7AAD staining **(D)** in 20% stretched cells pretreated with Thapsigargin to empty ER Ca^2+^ stores. n = 3 biologically independent samples. **(E)** Relative expressions of mRNA of Ca^2+^ receptors in 20% stretched cells detected by real time PCR. n = 3 biologically independent samples. **(F-G)** Representative histograms of Dextran 10K-FITC penetration **(F)** or 7AAD staining **(G)** in 20% stretched cells pretreated with RYR inhibitor Dantrolene. n = 3 biologically independent samples. Data are presented as the mean ± s.d. Statistical significance was assessed by one-way ANOVA with Tukey’s post hoc test **(A-D, F-G)** and unpaired two-tailed Student’s t-test **(E)**.


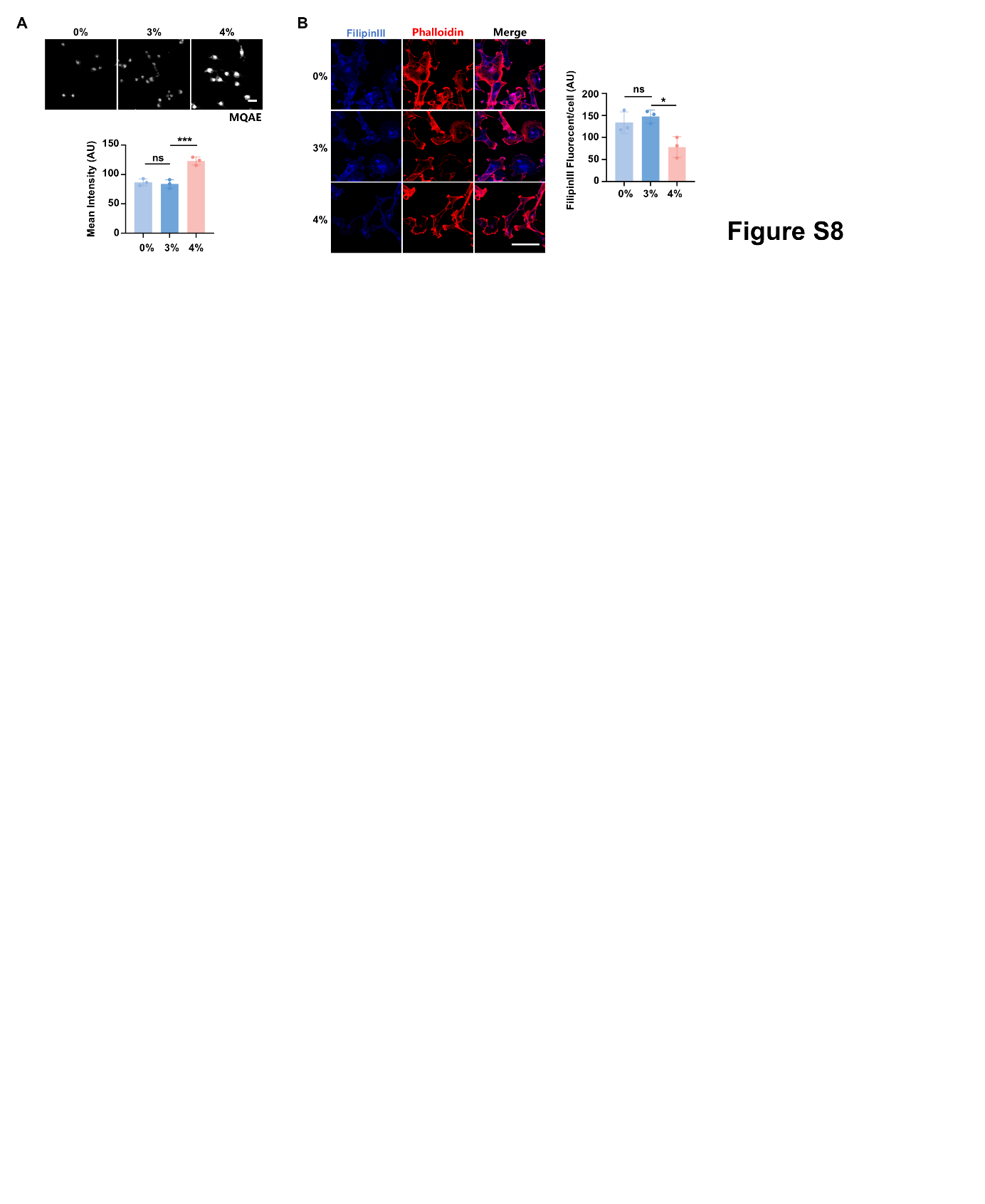


**Figure S8. VRAC activation and membrane cholesterol content in BEAS-2b under stretch**

**(A)** Representative fluorescence images of Cl^−^ sensor MQAE detected by CLSM and quantitative fluorescence intensity. Scale bar, 20 µm. n = 3 biologically independent samples. **(B)** Representative fluorescence images of cells stained with FilipinIII and quantitative fluorescence intensity. Scale bar, 20 µm. n = 3 biologically independent samples. Data are presented as the mean ± s.d. Statistical significance was assessed by one-way ANOVA with Tukey’s post hoc test **(A-B)**.


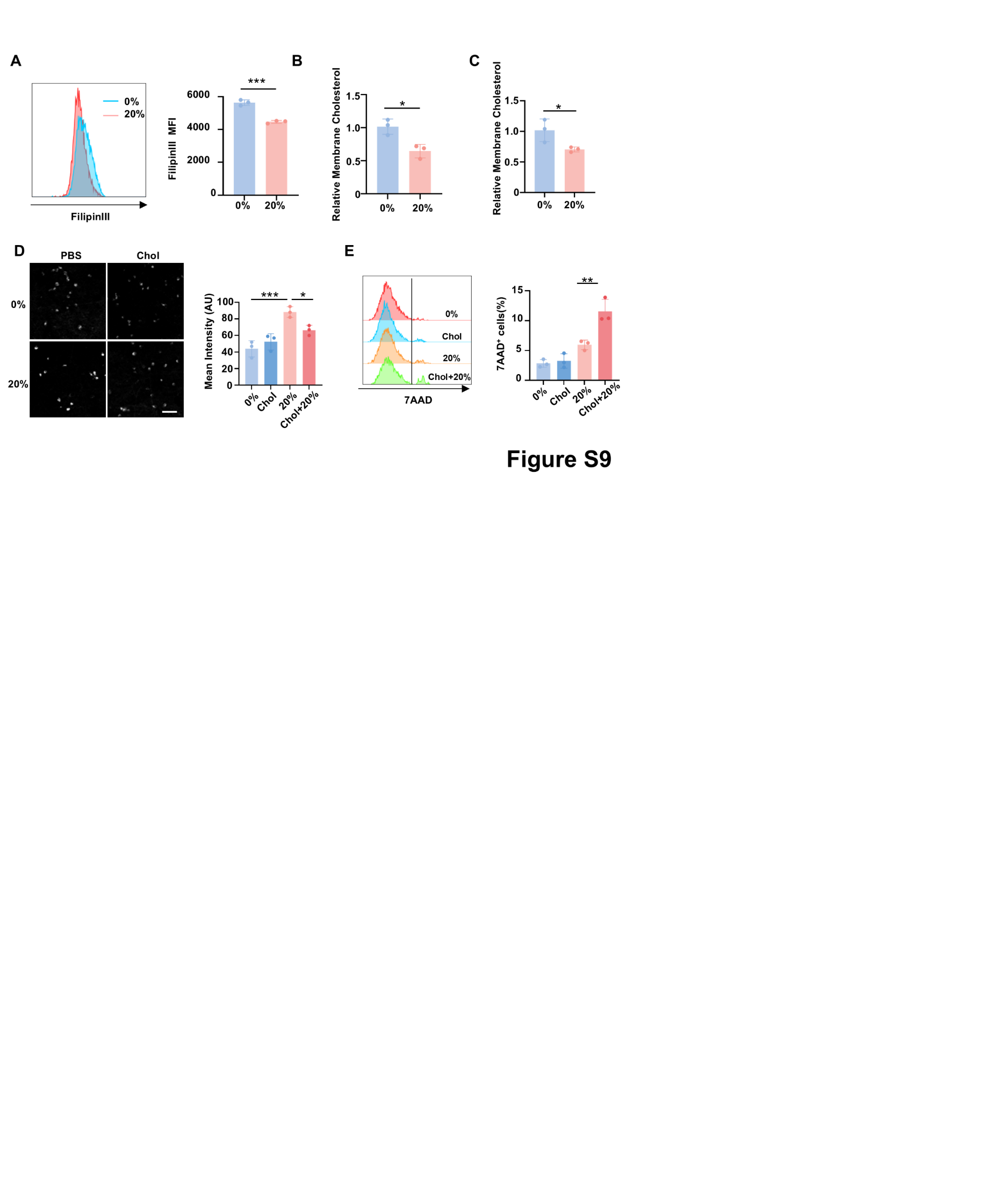


**Figure S9. Stretch-induced VRAC activation via membrane cholesterol efflux in alveolar epithelial cells**

**(A)** Representative histograms of Filipin III staining and quantification analysis. **(B)** Quantification of membrane cholesterol content by Amplite Cholesterol Quantitation Kit. **(C)** Membrane cholesterol content was quantified by subtracting intracellular cholesterol from total cellular cholesterol. **(D)** Representative fluorescence images and quantiﬁcation of MQAE intensity. Scale bar, 20 µm. **(E)** Representative histograms and quantiﬁcation analysis of 7AAD staining. Data are presented as the mean ± s.d. Statistical significance was assessed by one-way ANOVA with Tukey’s post hoc test **(D, E)** and unpaired two-tailed Student’s t-test **(A-C)**.


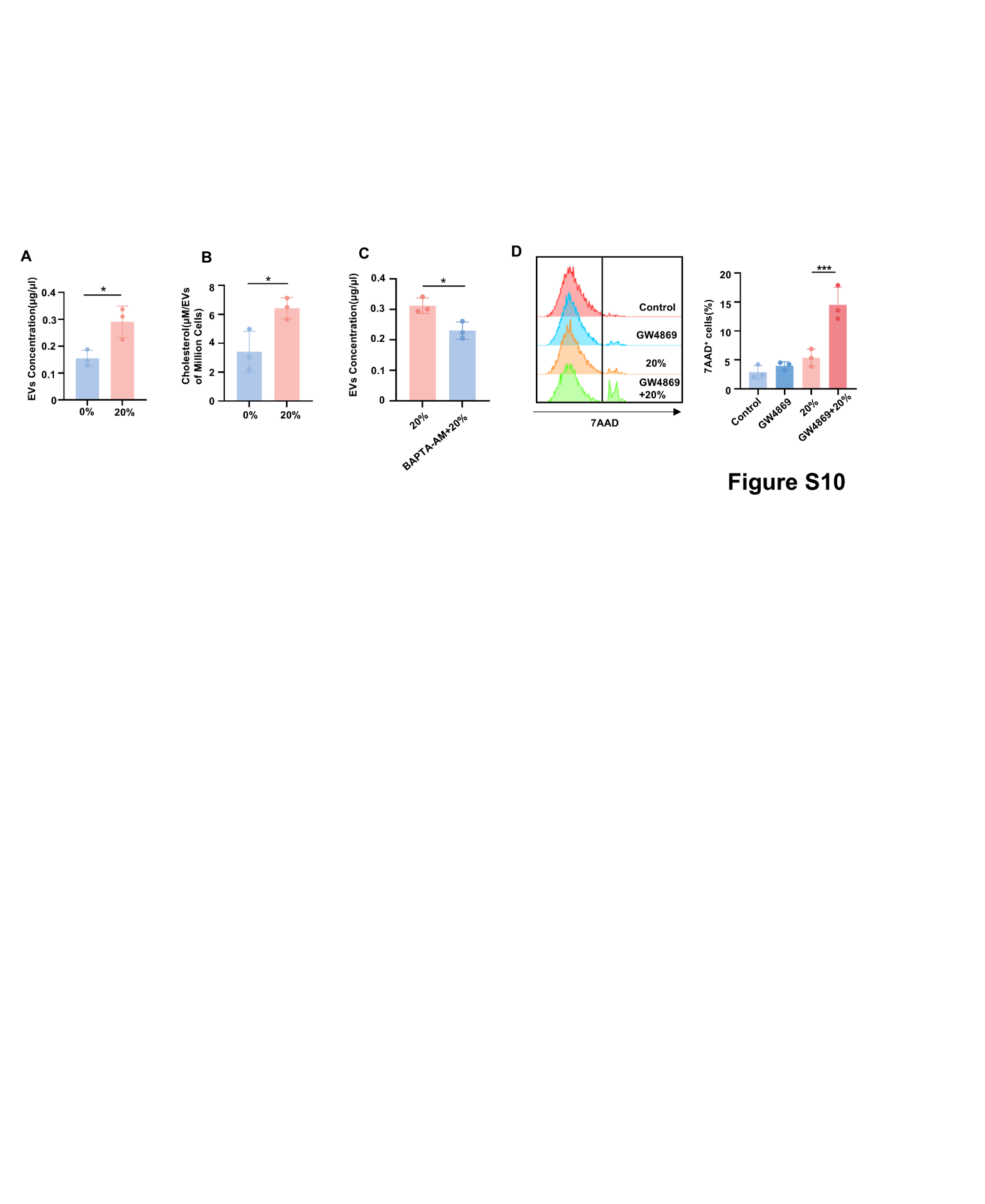


**Figure S10. Stretch-induced membrane cholesterol efflux mediated by Ca^2+^-dependent EVs release in alveolar epithelial cells**

**(A)** The concentration of EVs released from an equivalent number of cells, analyzed by BCA assay. n = 3 biologically independent experiments. **(B)** Cholesterol content in EVs derived from an equivalent number of cells. n = 3 biologically independent experiments. **(C)** The concentration of EVs released from an equivalent number of stretched cells with or without the pretreatment of BAPTA-AM. n = 3 biologically independent experiments. **(D)** Representative histograms of 7AAD staining and quantitative analysis. n = 3 biologically independent experiments. Data are presented as the mean ± s.d. Statistical significance was assessed by one-way ANOVA with Tukey’s post hoc test **(D)** and unpaired two-tailed Student’s t-test **(A-C)**.


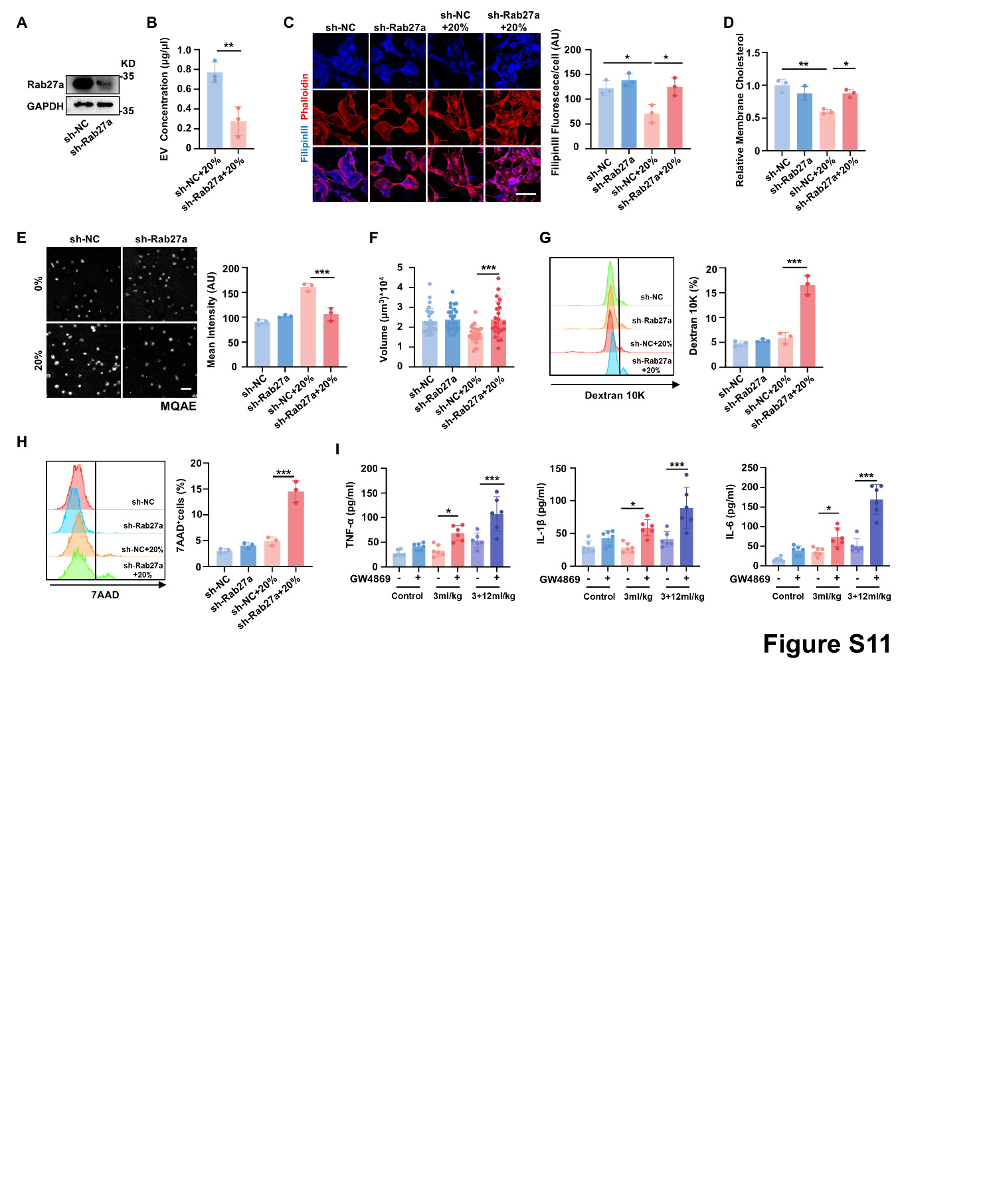
**Figure S11. The effect of EVs release on the emergence of mechanical adaptation**

**(A-H)** To knockdown Rab27a expression in BEAS-2b, cells were transfected with lentiviral vector encoding a short hairpin RNA-targeted Rab27a (sh-Rab27a), the same lentiviral vector carrying nonspecific RNA (sh-NC) was used as negative. **(A)** Western blot analysis of Rab27a expression in lentiviral transfected BEAS-2b. **(B)** Concentration of EVs released from stretched cells, detected by BCA analysis, n = 3 biologically independent samples. **(C)** Representative fluorescence images of FilipinIII staining and quantitative fluorescence intensity. Scale bar, 20 µm. n = 3 biologically independent experiments. **(D)** Quantification of membrane cholesterol content. n = 3 biologically independent experiments. **(E)** Representative fluorescence images and quantiﬁcation of MQAE intensity. Scale bar, 20 µm. n = 3 biologically independent experiments. **(F)** Cell volume analyzed by Imaris. n > 25 per group from three independent experiments. **(G-H)** Representative histograms of Dextran 10K-FITC **(G)** and 7AAD staining **(H)** in stretched cells and quantitative analysis to show cell damage. n = 3 biologically independent experiments. **(I)** Concentration of inflammatory factor TNF-α, IL-1β and IL-6 in BALF after ventilation detected by ELISA. n = 6 mice. Data were presented as the mean ± s.d. Statistical significance was assessed by one-way ANOVA with Tukey’s post hoc test **(C-I)** and unpaired two-tailed Student’s t-test **(B)**.


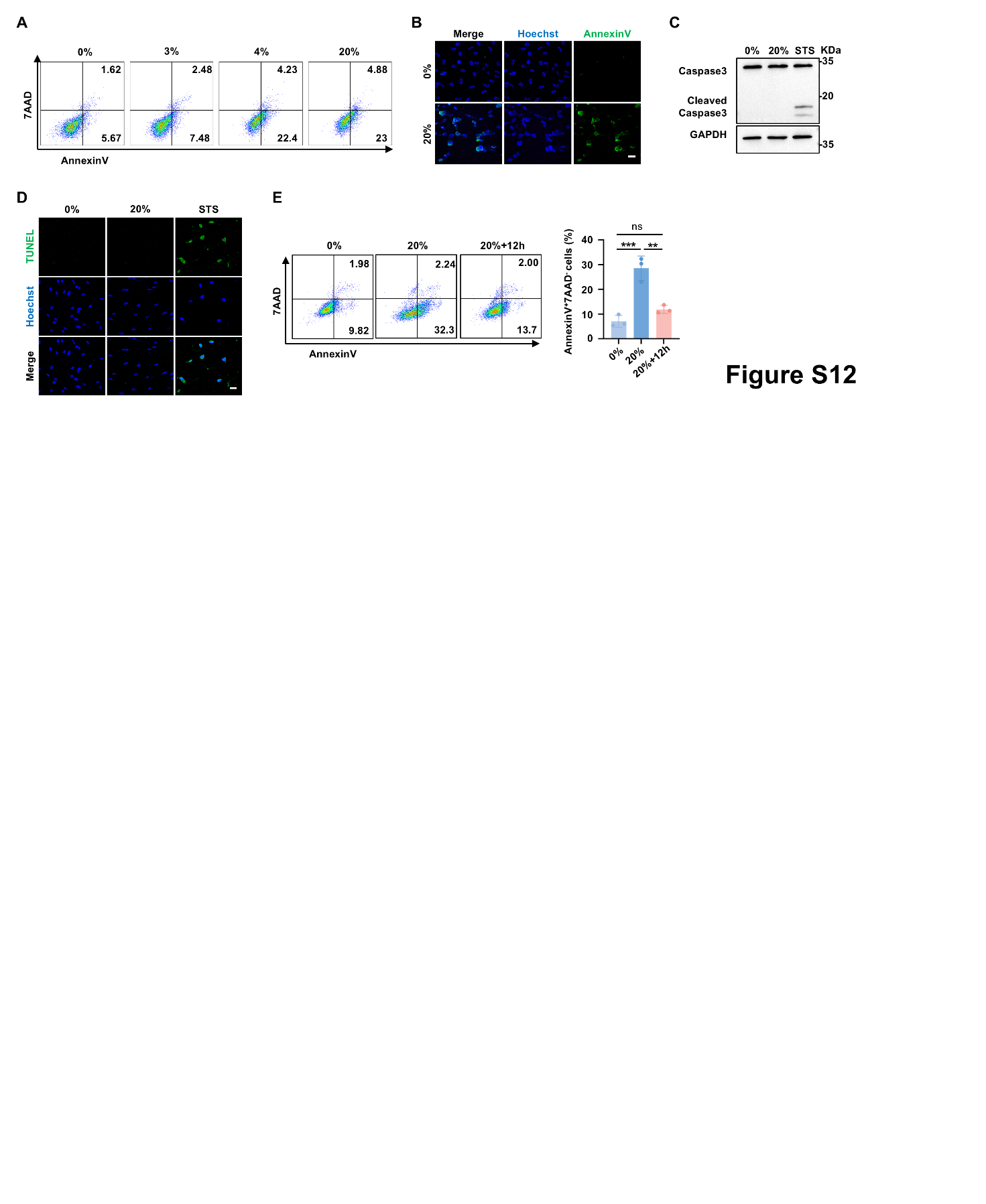


**Figure S12. Stretch-induced lipid scrambling did not accompany with cell apoptosis**

**(A)** Representative dot plots of AnnexinV and 7AAD staining in stretched cells detected by flow cytometry to analyze membrane lipid scrambling. **(B)** PS exposure of stretched cells analyzed through AnnexinV staining and detected by CLSM. Scale bar, 20 μm. **(C)** Western blot analysis of Caspase-3 and cleaved Caspase-3 in 20% stretched cells. Staurosporine (STS) served as a positive control to induce cell apoptosis. **(D)** Representative ﬂuorescence images of BEAS-2b cells stretched at 20% by TUNEL staining. Scale bar, 20 μm. **(E)** Representative dot plots and quantitative analysis of AnnexinV and 7AAD staining immediately after stretch or 12 hours post-stretch, detected by flow cytometry to evaluate lipid scrambling reversibility. n = 3 biologically independent experiments. Data were presented as the mean ± s.d. Statistical significance was assessed by one-way ANOVA with Tukey’s post hoc test **(E)**.


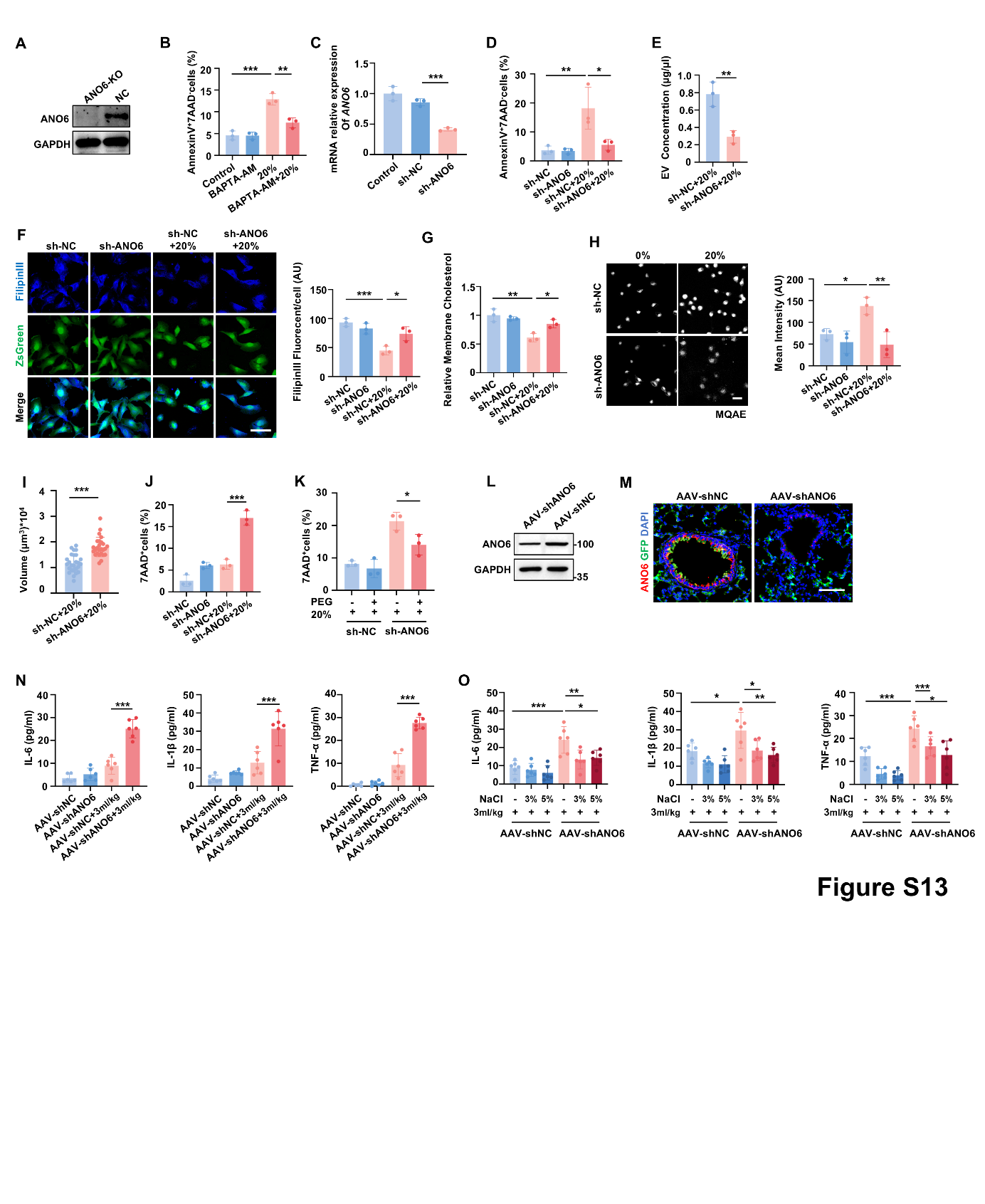


**Figure S13. The role of ANO6 in the emergence of mechanical adaptation**

(A) Western Blot analysis confirmed the ANO6 KO efficiency in alveolar epithelial cells. (B) Flow cytometry analysis of AnnexinV^+^7AAD^-^ cell percentage to assess membrane lipid scrambling in 20% stretched cells with or without pretreatment of BAPTA-AM. n = 3 biologically independent experiments. (C) *ANO6* relative expressions in lentiviral transfected BEAS-2b cells by real time PCR. (D) Quantitative analysis of AnnexinV^+^ 7AAD^-^ cell percentage detected by flow cytometry. n = 3 biologically independent experiments. (E) Concentration of EVs released from an equivalent number of 20% stretched cells. n = 3 biologically independent experiments. (F) Representative fluorescence images of cells stained with Filipin III and quantitative fluorescence intensity. Scale bar, 20 µm. n = 3 biologically independent experiments. (G) Quantification of membrane cholesterol content. n = 3 biologically independent experiments. (H) Representative fluorescence images of MQAE and quantiﬁcation of fluorescence intensity. Scale bar, 20 µm. n = 3 biologically independent experiments. (I) Cell volume analyzed by Imaris. n > 25 per group from three independent experiments. (J) Quantitative analysis of 7AAD^+^ cell percentage. n = 3 biologically independent experiments. (K) Quantitative analysis of 7AAD^+^ cell percentage in 20% stretched cells with or without 1% PEG300 pretreatment in the condition of ANO6 knockdown. n = 3 biologically independent experiments. (L) Western blot analysis of ANO6 expression in lung tissues. (M) Representative fluorescence images showed ANO6 (red) expressions in lung tissues. Scale bar, 100 μm. (N) Concentration of inflammatory factors TNF-α, IL-1β and IL-6 in BALF after ventilation detected by ELISA. n = 6 mice. (O) Concentration of inflammatory factors TNF-α, IL-1β and IL-6 in BALF after ventilation in the condition of NaCl hyperosmotic nebulization pretreatment detected by ELISA. n = 6 mice. Data are presented as the mean ± s.d. Statistical significance was assessed by one-way ANOVA with Tukey’s post hoc test (B-D, F-H, J, K, N, O), and unpaired two-tailed Student’s t-test (E, I)
